# Climate-Related Hazards: A Method for Global Assessment of Urban and Rural Population Exposure to Cyclones, Droughts, and Floods

**DOI:** 10.3390/ijerph110202169

**Published:** 2014-02-21

**Authors:** Elizabeth Christenson, Mark Elliott, Ovik Banerjee, Laura Hamrick, Jamie Bartram

**Affiliations:** 1 The Water Institute, Gillings School of Global Public Health, University of North Carolina, Chapel Hill, NC 27516, USA; E-Mails: cgelizab@email.unc.edu (E.C.); laura.renee.hamrick@gmail.com (L.H.); 2 Department of Civil, Construction and Environmental Engineering, University of Alabama, Tuscaloosa, AL 35406, USA; E-Mail: melliott@eng.ua.edu

**Keywords:** climate change, hazard events, exposure, ArcGIS, urban, rural, cyclone, drought, flood

## Abstract

Global climate change (GCC) has led to increased focus on the occurrence of, and preparation for, climate-related extremes and hazards. Population exposure, the relative likelihood that a person in a given location was exposed to a given hazard event(s) in a given period of time, was the outcome for this analysis. Our objectives were to develop a method for estimating the population exposure at the country level to the climate-related hazards cyclone, drought, and flood; develop a method that readily allows the addition of better datasets to an automated model; differentiate population exposure of urban and rural populations; and calculate and present the results of exposure scores and ranking of countries based on the country-wide, urban, and rural population exposures to cyclone, drought, and flood. Gridded global datasets on cyclone, drought and flood occurrence as well as population density were combined and analysis was carried out using ArcGIS. Results presented include global maps of ranked country-level population exposure to cyclone, drought, flood and multiple hazards. Analyses by geography and human development index (HDI) are also included. The results and analyses of this exposure assessment have implications for country-level adaptation. It can also be used to help prioritize aid decisions and allocation of adaptation resources between countries and within a country. This model is designed to allow flexibility in applying cyclone, drought and flood exposure to a range of outcomes and adaptation measures.

## 1. Introduction

Global climate change (GCC) has led to increased focus on the occurrence of, and preparation for, climate-related extremes and hazard events. The first step to evaluating the vulnerability of a country to climate-related hazard events is to establish a baseline for population exposure to these hazard events. The Intergovernmental Panel for Climate Change (IPCC) [1] indicates that increases in temperature, intensity of precipitation extremes, rainfall intensity, storm intensity and variability are likely or extremely likely as a result of GCC. Although international efforts to model likelihood of hazard events have been influential in country prioritization for emergency lending or grants and placement of disaster reduction advisors [2], these models often assess the likelihood of a specific outcome (e.g., mortality) and thus may not be directly applicable to other outcomes (e.g., loss of drinking water access). This research used previously-applied approaches to disaster risk assessment to develop a population exposure assessment specific to climate-related hazard events that can be broadly applied to specific outcomes of interest. This introduction describes three influential global models, their relative strengths and weaknesses compared to the one we present, and our objectives.

### 1.1. Previous Exposure Assessments

Three global studies of population risk assessments of hazard events have influenced policy in countries worldwide [2]: the Disaster Risk Index (DRI) by the United Nations Development Program (UNDP) [3]; the Global Natural Disasters Risk Hotspots (GNDRH) developed by Columbia University, the World Bank, and the Norwegian Technical Institute [4]; and the biennial global assessment reports (GAR) by the United Nations International Strategy for Disaster Reduction (UNISDR) [5,6,7]. These risk assessments all estimate physical exposure and the resulting mortality associated with hazard events including cyclone, drought, earthquake, flood, and landslide. Physical exposure is a measure of the population-weighted “frequency and or (sic) the probability of hazard events at each location” [2]; it corresponds to the likelihood that a person in that location would be exposed to (a) given hazard event(s) in a given period of time. The previous risk assessments are based in the theory that loss is dependent on the frequency of the hazard, the number of people exposed, and the vulnerability of people to a hazard. The methods used in these assessments are summarized and compared below; methods are described in the original publications [3,4,5,6,7].

The DRI [3] estimates mortality on a country level by first calculating likelihood of physical exposure by multiplying population in a location by relative hazard frequency data for cyclones, droughts, floods (including floods caused by landslides), and earthquakes. DRI weights population exposure using a regression equation, using variables associated with each hazard type to match observed mortality. After calculating vulnerability at a location, represented by a grid cell, these cell values are aggregated (summed) over the entire country to rank relative risk among countries. 

Like DRI, GNDRH [4], GAR 2009 [5], and GAR 2011 [6] estimate mortality due to hazard events; in addition, they also estimate economic loss. Both GNDRH and GAR estimate mortality in a population using mortality data and relative hazard frequency data. Whereas DRI estimates mortality in a population at the time of the hazard event, GNDRH aggregates all historical hazard data to estimate cumulative mortality in the current population. GNDRH mortality is estimated based on population exposure to each hazard event multiplied by “vulnerability coefficients” that are based on region of the world and wealth status. The largest difference between DRI and GNDRH is the method used to determine vulnerability [2]. DRI accounts for spatial and social characteristics of the setting by using different variables to describe vulnerability for each hazard event, but the estimate depends on reported mortality data. The GNDRH method accounts for differences among regions using wealth status but does not account for differences in vulnerability to specific hazard events. Similar to DRI, GAR identifies variables that best explain likelihood of mortality to different types of hazards. The latest GAR release in 2013 [7], however, applies a probabilistic approach to estimating mortality but focuses primarily on improving their model for vulnerability to economic loss.

DRI, GNDRH, and GAR estimate mortality for each hazard event using different datasets and methods of compiling population and relative hazard frequency data. They display relative likelihood of mortality for each grid cell in deciles for each hazard and sum the likelihoods of all hazards to determine multi-hazard likelihood of mortality. Finally, the values in each grid cell are aggregated for a country to calculate the estimated mortality of the country for each hazard event. Table S1 in the Online Supplementary Information contains summary comparisons of DRI, GNDRH, and GAR.

These risk assessments have been instrumental in aggregating, modeling, and refining global hazard event occurrence data and have been invaluable for identifying countries with high likelihood of mortality and economic loss due to hazard events. However, we have identified two important characteristics of these models that prevent their application to some outcome variables.

These models have estimated the likelihood of a certain outcome (e.g., likelihood of mortality or economic loss) of populations rather than exposure. Directly assessing the likelihood of outcomes such as mortality conflates exposure to the hazard event with susceptibility and adaptive capacity. Separating exposure allows model results to be applied more specifically to the vulnerability being measured, using vulnerability variables tailored to specific outcomes. The methods used by DRI, GNDRH, and GAR to model exposure to an adverse outcome (e.g., to mortality or to economic loss) limit the application of these models. For example, when assessing the vulnerability of community evacuation management procedures during flooding, transportation parameters will be paramount; in contrast, transportation parameters are not as relevant when assessing the vulnerability of drinking water supply to interruption or contamination. Additionally, separating out an exposure model allows uncertainty in the model to be more readily understood and avoids the shortcomings of using reported mortality and economic loss such as estimation errors and inconsistencies in data quality between countries.

Furthermore, these models have not disaggregated exposure between urban and rural populations. Separation of urban and rural exposure allows different socio-economic and other variables to be applied in resilience assessments based on the specific risk factors and adaptive capacities of urban and rural populations. Typical differences include easier access to multiple transportation options for disaster response, greater access to health care as well as other resources and expertise. On the other hand, emergency evacuation notices can be more difficult to execute in densely populated urban areas because transportation routes become congested [8] and the urban poor are much more susceptible to rising food prices than the rural poor [9]. The urban and rural poor have different vulnerabilities to climate-related hazards. The urban poor have higher risks associated with inequality in access to services, poor infrastructure, and increasing reliance on cash income. The rural poor, on the other hand, have risk factors associated with decreased access to services, the market, and transportation, as well as a reliance on subsistence agriculture [5,9,10].

### 1.2. Objectives

The objectives of this work were to: (a) develop a method for estimating the relative population exposure (the likelihood that a person in a location would be exposed to: given hazard event(s) in a given period of time) of countries to the climate-related hazard events of cyclone, drought, and flood; (b) develop a method that would readily allow the addition of updated and refined datasets to an automated model; (c) differentiate exposure of urban and rural populations to allow appropriate variables to be applied to these populations; (d) and present the results of a baseline ranking of countries based on the country-wide, urban, and rural population exposure to cyclone, drought, and flood.

## 2. Methods and Materials

### 2.1. Data Sources and Approach

Exposure is conceptualized here as the likelihood that an individual in a given location is exposed to a given type of climate-related hazard event over a certain period of time. Estimating population exposure to hazard events requires gridded datasets of population density and of the relative frequency (likelihood) of climate-related hazard events. The data sources used in our model were: population density global grid using LandScan^TM^ 2008 (this product was made utilizing the LandScan^TM^ (2009) High Resolution Global Population Data Set copyrighted by UT-Battelle, LLC, operator of Oak Ridge National Laboratory under Contract No. DE-AC05-00OR22725 with the United States Department of Energy. The United States Government has certain rights in this Data Set. Neither UT-Battelle, LLC nor the United States Department of Energy, nor any of their employees, makes any warranty, express or implied, or assumes any legal liability or responsibility for the accuracy, completeness, or usefulness of the data set) [11] and likelihood of hazard event for cyclones, droughts, and floods from Columbia University’s Center for Hazards and Risk Research (CHRR) [12,13,14].

Our model classifies each grid cell as urban or rural. Urban and rural population proportions for each country from the United Nations were used to identify an urban population density threshold for each country [15]. The analyses described in the subsections were carried out for all countries. The country of Thailand is used here as an illustrative example.

### 2.2. Population Density

The major publically available gridded global population datasets: Gridded Population of the World version 3 (GPW) [16], Global Rural–Urban Mapping Project version 1 (GRUMP) [17], and LandScan^TM^ 2008 [11] were reviewed to determine which was best suited for this analysis. These datasets use different population data, ancillary data, and interpolation methods to generate a grid of global population (Table 1).

**Table 1:** Comparison of modeled global population datasets ^1^.

Dataset	Spatial Resolution	Years	Census Input Data	Ancillary Variables	Interpolation Method	Source
GPW v3	2.5' × 2.5' (~5 × 5 km)	1990, 1995, 2000; projected to 2005, 2010, 2015	UNDP	N/A	Areal weighting across administrative area	Balk *et al*. 2004 [18]
GRUMP v1	0.5' × 0.5' (~ 1 × 1 km)	1990, 1995, 2000	UNDP	Nighttime lights, populated areas	Areal weighting is reallocated based on urban extent ^**2**^ within an administrative area.	Balk *et al*. 2005 [19]
LandScan^TM^	0.5' × 0.5' (~1 × 1 km)	annual	CIA	Roads, Slope, Elevation, Land Cover, Populated areas, Nighttime lights	Smart interpolation reallocates population within an administrative area based on the probability coefficient of the grid cell. ^**3**^	Dobson *et al*. 2000 [20]

Notes: ^1^ Adapted from Tatem *et al*. [21] and Galway *et al*. [22]; ^**2**^ Urban extent modeled using night-time light estimates and settlement locations; **3** Probability coefficient predicts how likely a grid cell will be inhabited based on LandScan^TM^ ancillary variables.

**Figure 1:**
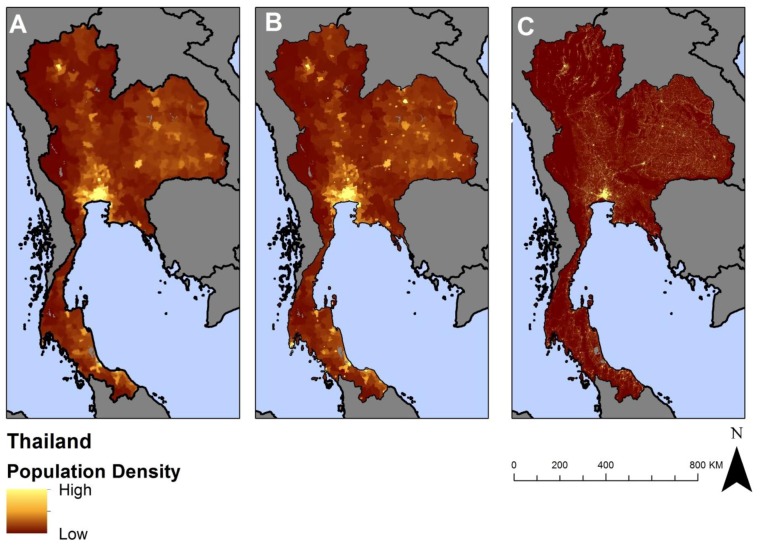
Visualization of three global population density datasets using Thailand as an example (A) Gridded Population of the World version 3 (GPW v3); (B) Global Rural–Urban Mapping Project version 1 (GRUMP v1) and (C) LandScan^TM^.

The LandScan^TM^ 2008 dataset was chosen because of its frequent updates and interpolation method. LandScan^TM^ updates annually with recent census data as it becomes available as well as ancillary data used for the model; this will be important if our method is used to evaluate changes over time due to climate change, whereas GPW was most recently released in 2000 and projected to the future at five-year intervals (e.g., 2005, 2010, 2015). Although GRUMP was published in 2011, it estimates population using ancillary data from 2000. Additionally, the interpolation method varies between each dataset for estimating population density in a location. GPW uses an “areal weighting” that distributes census population proportionally across each administrative area (see Figure 1A). GRUMP updates GPW’s approach and distributes more of an administrative area’s population to GRUMP-defined urban areas (Figure 1B). In contrast, the LandScan^TM^ interpolation method redistributes input census population data with ancillary data to estimate population distribution within administrative units (Figure 1C). This difference in methodology is important at the sub-national level [21].

The major disadvantage of LandScan^TM^ is that the method is less transparent than the open source methods used by GPW and GRUMP [21,22,23]. LandScan^TM^ lacks complete transparency in the specific algorithms used to model population and in the documentation of spatial and temporal resolution of the input census data for each country. These limitations prevent researchers from analyzing error and uncertainty in the underlying data sources. However, both the core population dataset and the ancillary variables used in the model are publically available.

### 2.3. Climate-Related Hazard Events

Data on the occurrence of hazard events are available for download from the Columbia University Center for Hazards and Risk Research (CHRR) [12,13,14]. These data were generated through collaboration between CHRR, the Norwegian Geotechnical Institute (NGI) and Center for International Earth Science Information Network (CIESIN). Datasets include global grids of the frequency of cyclone, drought, earthquake, flood, landslide, and volcano frequency and distribution. We include cyclones, droughts, and floods into this exposure assessment because they are related to climate. Volcanoes and earthquakes have not been shown to correlate with climate. Although there is evidence to indicate that slope stability is directly impacted by climate change [24,25,26,27] disaggregating landslide events caused by seismic activity or human-induced activity such as deforestation confound the landslide frequency data available. Crozier argues that landslide frequency may even be dominated more by human activity than climate-induced weakened slopes [28].

The probability of cyclone, drought, and flood occurrence vary geographically. A dataset of reported recent occurrences of these events (between approximately 1980 and 2000) was used by CHRR to create a global grid reflecting the likelihood of each hazard event at that grid cell. CHRR excluded cells “that had a population density less than 5 persons per square kilometer and without significant agriculture” [4].

**Figure 2:**
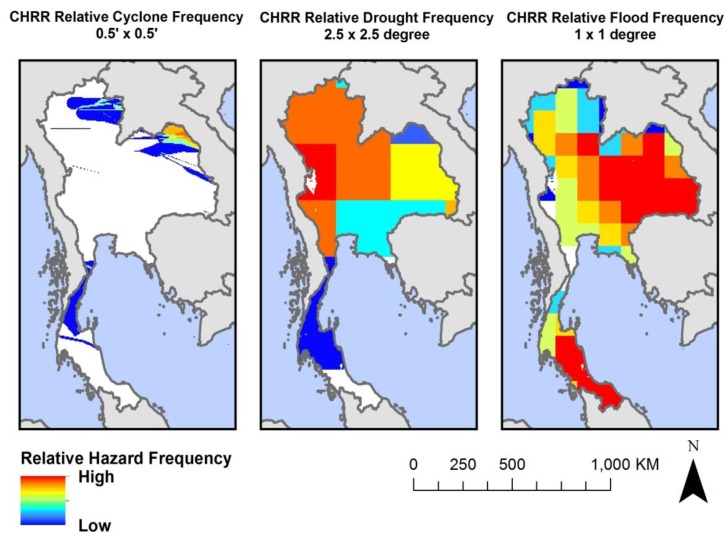
Relative cyclone, drought and flood frequency deciles based on global CHRR hazard data for Thailand [12,13,14]. White areas in Thailand represent locations where no hazard events were reported by CHRR over the period of analysis.

CHRR placed each grid cell with sufficient population and agriculture into a decile reflecting the relative frequency of each hazard event at that grid cell. CHRR presents their data as deciles instead of absolute frequency to represent 20-year total relative likelihood of hazard occurrence “to avoid literal interpretation” and “in recognition of the many limitations of the underlying data” [4]. We used these deciles as proxies for a baseline relative probability of cyclone, drought, and flood at each grid cell on the globe. An example illustration is provided in Figure 2.

Cyclone relative frequency and distribution data by CHRR are based on more than 1,600 historic storm tracks for 1980–2000 (Table 2). These events included tropical storms, hurricanes, typhoons, tropical depressions, and other large tropical wind events. The storm track data was assembled at UNEP/GRID-Geneva PreView into a global 0.5' × 0.5' grid. To determine the geographic extent likely to have been affected by each event, a Holland wind speed model was used to create buffer extents based on the six wind speed categories of the Saffir-Simpson Hurricane scale. Cells within the storm track’s buffer extent were counted as having had a cyclone event. Thus, cyclone relative frequency reflects high wind rather than storm surges or other flooding that are secondary effects associated with cyclones. For each grid cell that was part of the storm track extent, the cell received a tally. Frequency of cyclone event was determined by summing the number of cyclone events that affected each grid cell. All cells were then sorted into deciles representing the relative frequency of cyclone events from 1980–2000.

**Table 2:** Summary of CHRR hazard relative frequency data sources used to calculate exposure.

Data Type	Data Source	Units	Spatial Resolution	Time Period	Description
Relative Cyclone Frequency	CHRR	Frequency	0.5' × 0.5'(~1 × 1 km) 1,600 events	1980–2000	UNEP/GRID—Geneva PreView uses Saffir Simpson Hurricane index determines extent
Relative Drought Frequency	CHRR	Frequency	2.5° × 2.5° (~276 × 276 km) Unspecified number of events	1980–2000	IRI Climate Data Library uses drought defined by WASP
Relative Flood Frequency	CHRR	Frequency	1° × 1° (~111 × 111 km) 3,700 events	1985–2003	Dartmouth Flood Observatory uses satellite imagery to determine extent of extreme flood events

Drought likelihood data from CHRR are based on International Research Institute for Climate Prediction’s (IRI) Weighted Anomaly of Standardized Precipitation (WASP), which collected average monthly precipitation from 1980–2000 for a global 2.5° × 2.5° grid (Table 2). IRI collaborated with CHRR and CIESIN to identify drought events. IRI, CHRR and CIESIN defined a drought event as when the magnitude of monthly precipitation was less than or equal to 50 percent of its long term median value for three or more consecutive months [4]. They then sorted all cells based on relative frequency of drought events over the 1980–2000 period and sorted the cells into deciles.

Flood likelihood data from CHRR are based on over 3,700 extreme flood events between 1985 and 2003 (Table 2). Floods may be due to heavy rain, cyclone-type events, snowmelt, ice jams, dam failures, or avalanches. Flood inundation events were compiled by the Dartmouth Flood Observatory using satellite imagery and georeferenced to the nearest degree. Not all flooding events are climate-related (e.g., avalanche-related flooding). However, our review of the data indicated that floods caused by climate-unrelated events were less than 1% of the total number of flooding events. Therefore all flood events used by CHRR were retained in our analysis. Flood frequency was defined by the number of times a grid cell was inundated over the period of analysis rather than the number of days a grid cell was inundated. For each grid cell that contained a flood event, the cell received a tally. Frequency of flood event was determined by summing the number of flood events that affected each grid cell. All grid cells were sorted into deciles representing the relative frequency of flood events from 1985–2003.

### 2.4. Calculating Average Exposure of Urban and Rural Populations

Previous spatial identification of urban extent has primarily relied on two general methods: satellite imagery and ground inputs (see Potere *et al*. [29] for comparison of urban extent mapping). Methods vary widely and numerous data categories have been used in defining urban extent. These include: using satellite imagery of infrastructure including built up locations [30,31], impervious surfaces [32], and/or night-time lights [19,33] to measure urban extent. Ground input data include census estimates [16] and using an urban population density threshold [34,35,36].

Our model uses United Nations (UN) urban and rural population data, along with the LandScan^TM^ population density grid, to create an urban population density threshold for each country. Because there is no globally consistent definition of urban areas, the UN relies on nationally reported definitions of urban areas, which are based on a variety of parameters. Using national urban definitions is likely to better represent the urban character of a country than a global uniform standard. Where one country may solely define urbanity based on available infrastructure (e.g., existence of paved streets or water supply systems), another may define urbanity by population density, livelihoods, economic characteristics, and/or administrative boundaries [15]. The UN World Urbanization Prospects (WUP) [15] is considered the authoritative source for actual and projected urban and rural populations [35]. We used WUP estimates of urban and rural population in tabular form and the LandScan^TM^ population distribution map to create an urban population density threshold for each country that we then used to define each population grid cell as urban or rural. The application of this population density threshold model to estimate urban and rural exposure to climate-related hazards can use the best available population data to map urban and rural extent. However, the application of the model to future population exposure to hazard events due to GCC requires a population dataset that is projected into the future. The UN WUP has made long-term urban and rural population projections available. Therefore, our objective to generate a model that can be updated readily is compatible with UN data sources for population.

Additionally, defining urban areas by population density improves model applicability to urban- or rural-specific vulnerability to outcomes. For example, using measures of government services or building infrastructure to define urban extent and then using a measure of infrastructure (e.g., availability of piped drinking water) to analyze vulnerability of urban and rural areas to loss of water access could result in conflating or double-counting the input variables and outcomes.

To calculate the country-level urban population threshold, the LandScan^TM^ population grid was summed over an entire country and multiplied by the WUP proportion urban. The population value in each grid cell of the country was sorted and a threshold was determined to match the UN proportion urban. Grid cells above the population threshold were reclassified as urban grid cells and all others were reclassified as rural grid cells.

### 2.5. Calculating Population Exposure

Population exposure scores for each grid cell were calculated separately for cyclone, drought and flood. The population of each cell (*Pop_cell_*) was multiplied by the hazard event likelihood for that cell (*H*) according to Equation (1):

*Cell Exposure_drought_ = Pop_cell_ × H_drought,cell_* (1)

Individual hazard event exposures were summed to generate a multi-hazard cell exposure value (Equation (2)). The three types of hazard events were weighted equally:

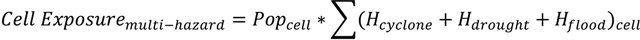
(2)


**Figure 3:**
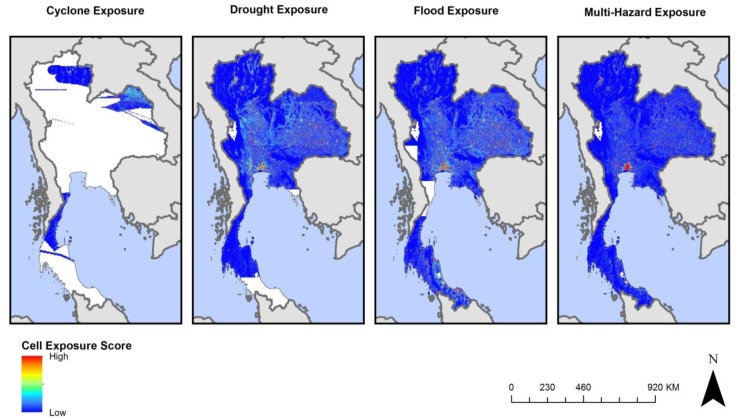
Cyclone, drought, flood, and multi-hazard cell exposure scores for Thailand. The concentration of high exposure (red) in Bangkok represents higher population exposure due to the concentration of population in that area, not necessarily higher population exposure. White areas in Thailand represent locations where no hazard events were reported by CHRR in the period of analysis.

An example of cell exposure for each hazard event and for the aggregated total for Thailand is displayed in Figure 3.

Country-level population exposure values were obtained by dividing by the total population of the country, as described below.

The cell exposure values were summed over urban locations, rural locations, and over the entire country to calculate the average population exposure scores for each country for both the multi-hazard exposure and individually for cyclone, drought, and flood. Country-level cyclone, drought, and flood exposure values have a theoretical maximum value of 1.0 (representative of the entire population of the country exposed to the highest hazard relative frequency decile). The total average population exposure was calculated as the sum of the cell exposure value of all cells in a country divided by the total population of all cells in the country (Equation (3)). The theoretical maximum, for total average population exposure is 3.0. Exposure was calculated likewise for the rural and urban areas in each country (Equations (4) and (5), respectively). These values were calculated for all hazard event types in aggregate (multi-hazard) and also individually for cyclone, drought and flood:

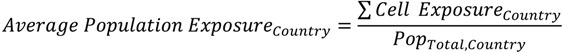
(3)

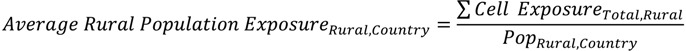
(4)

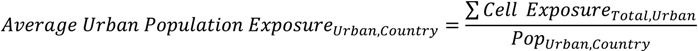
(5)


Country level scores were used to compare and rank countries based on average population exposure to cyclone, drought and flood individually and also the multi-hazard population exposure. Urban and rural average population exposure scores can be used to compare the average exposure of urban and rural populations both between countries and within a country.

### 2.6. Automated Model Development

The addition of new datasets, including global grids of population density and hazard events, was developed using ArcGIS 10.1 Model Builder [37,38] and Python 2.6 coding. This will allow assessment of changes in exposure over time and easy replacement of an existing dataset with a newly released or superior dataset. The model that automates exposure calculations has been made available on the UNC Water Institute website as an ArcGIS toolbox [39].

## 3. Results and Discussion

Data generated included population exposure scores for cyclone, drought, flood and multi-hazard. These scores were generated for all 228 countries and territories for urban, rural and total populations. These data are presented in total in Table S2 of the Online Supplementary Information.

Results presented in this section are in the form of rankings of population exposure at the country-level. In maps, these are displayed as country-level population exposure with equal intervals (red = highest quintile of population exposure). In tables, these are displayed as population exposure rankings (1 = country with highest population exposure, 2 = second highest population exposure, *etc*.). Note that the populations of some countries were not exposed to a given hazard event over the 1980–2000 or 1985–2003 periods of analysis, resulting in many countries tying in the ranking for “least exposure”. This is especially striking for cyclone exposure, a hazard that is highly concentrated in certain regions, in which the majority of countries (136 of 228) were not exposed to a cyclone during the 1980–2000 period of analysis. Therefore, the country rankings represented on the maps may have the highest rank representing many countries tied for the highest rank (*i.e.*, least exposure) (see Tables S3–S6 in the Online Supplementary Information).

We also compared country exposure within and across the Human Development Index (HDI) categories [40]. The 2012 HDI equally splits 187 countries into four development classes (Low, Medium, High and Very High) based on the country’s level of education, gross national income per capita, and life expectancy at birth. Very high HDI countries are the most developed and generally the wealthiest. Table 3 below compares HDI classes with global average cyclone, drought, and flood country-level, urban, and rural population exposure.

### 3.1. Cyclone Country-level Population Exposure

**Figure 4:**
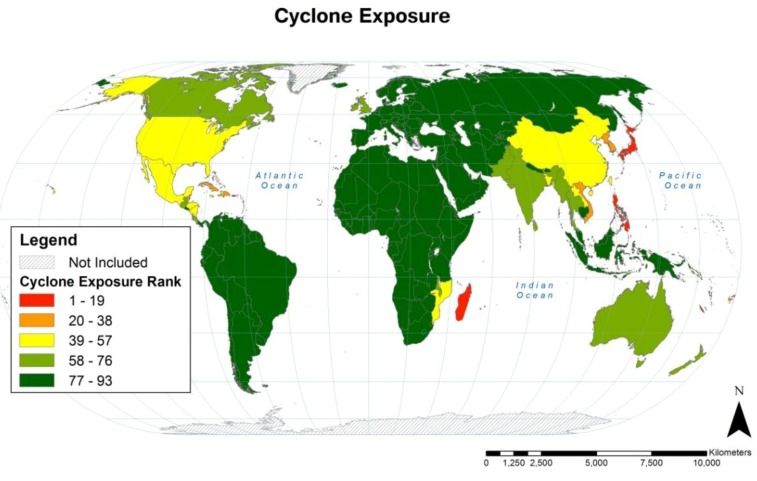
Rankings of country-level population cyclone exposure expressed as quintiles. The majority of countries (136 of 228) had no cyclones in populated areas during the 1980–2000 period of analysis and are all tied for 93rd rank.

Country-level rankings of population exposure to cyclone are illustrated in Figure 4 and the rankings of the countries with the greatest population cyclone exposure are listed in Table 4.

**Table 3:** Comparison of global and HDI class average population exposure for cyclone, drought, flood and multi-hazard for total, urban, and rural populations.

HDI	Global	Very High	High	Medium	Low
**Cyclone**	**0.12**	0.21	0.02	0.14	0.05
Urban Cyclone	**0.15**	0.22	0.02	0.19	0.04
Rural Cyclone	**0.09**	0.13	0.02	0.1	0.05
**Drought**	**0.37**	0.14	0.27	0.46	0.5
Urban Drought	**0.32**	0.14	0.27	0.43	0.53
Rural Drought	**0.43**	0.11	0.27	0.49	0.47
**Flood**	**0.51**	0.48	0.4	0.57	0.47
Urban Flood	**0.51**	0.49	0.41	0.59	0.48
Rural Flood	**0.5**	0.42	0.36	0.55	0.47
**Multi-Hazard**	**1**	0.82	0.68	1.17	1.02
Urban Multi-Hazard	**0.98**	0.86	0.69	1.21	1.05
Rural Multi-Hazard	**1.02**	0.66	0.65	1.14	0.99

**Table 4:** Ranked lists of the twenty countries with the greatest population exposure to cyclones, droughts, and floods.

Country	Cyclone Rank	Country	Drought Rank	Country	Flood Rank
Guam	1	Gibraltar	1	Macao	1
Northern Mariana Islands	2	Lebanon	2	Bangladesh	2
Réunion	3	Malta	2	Hong Kong	3
Mauritius	4	Nauru	2	Jamaica	4
Hong Kong	5	Swaziland	2	Guatemala	5
New Caledonia	6	Saint Kitts and Nevis	6	Nepal	6
Japan	7	Djibouti	7	Liechtenstein	7
British Virgin Islands	8	Jordan	8	Singapore	7
Antigua and Barbuda	9	Myanmar	9	El Salvador	9
Macao	10	Guatemala	10	Honduras	10
Philippines	11	Syria	11	Sri Lanka	11
Madagascar	12	Zimbabwe	12	Vietnam	12
United States Virgin Islands	13	Eritrea	13	Haiti	13
Puerto Rico	14	Pakistan	14	Cambodia	14
Montserrat	15	Somalia	15	South Korea	15
Anguilla	16	Lesotho	16	Colombia	16
Guadeloupe	17	Iraq	17	Ecuador	17
Vanuatu	18	Chile	18	Kenya	18
Dominica	19	Kiribati	19	Rwanda	19
Saint Kitts and Nevis	20	Malawi	20	Thailand	20

Cyclone exposure was highest in five geographic regions during the period of analysis: (1) East Asia and Southeast Asia; (2) North America, Central America and the Caribbean; (3) southeastern Africa, Madagascar and the surrounding islands; (4) South Asia; and (5) Pacific Island countries. Populations of some other countries outside these regions (e.g., United Kingdom, Russia and Australia) were also exposed to cyclone, but the majority of countries (136 of 228) had no cyclones in populated areas during the 1980–2000 period of analysis and all rank 93rd in cyclone exposure. Cyclone exposure by HDI class was highest for very high HDI countries, 71% above the global average, and lowest for high HDI countries, 84% below the global average (see Table 3).

### 3.2. Drought Country-level Population Exposure

**Figure 5:**
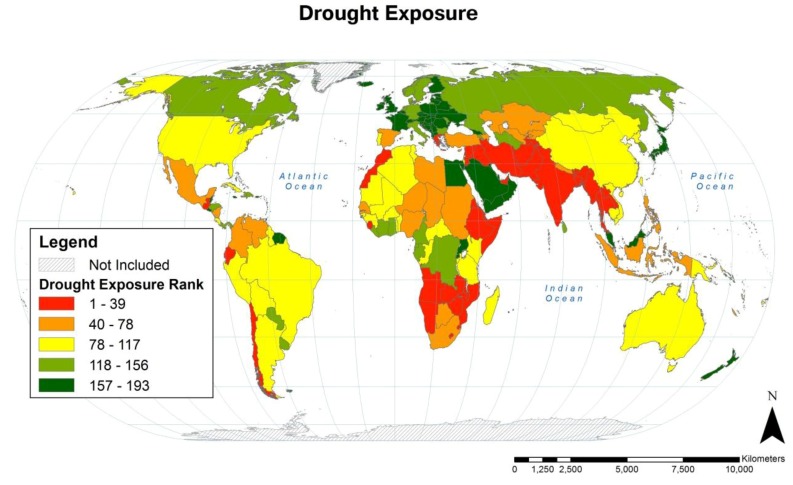
Rankings of country-level population drought exposure expressed as quintiles. Thirty-six countries (of 228) had no droughts in populated areas during the 1980–2000 period of analysis and are each tied for 193rd rank.

Country-level rankings of population exposure to drought are illustrated in Figure 5 and the rankings of the countries with the greatest population drought exposure are listed in Table 4.

Drought exposure is more broadly distributed than cyclone exposure. Populations with high drought exposure during the 1980–2000 period of analysis include those of most countries in South Asia, Southeast Asia, and Western Asia through to the Mediterranean.. Populations of most African countries also had moderate to high exposure to drought, as did those living in some countries of the Americas and Pacific Islands.

Drought was defined in our data source as a precipitation amount less than half its median three consecutive month value [4]. This definition, based on deviation from historical precipitation patterns, created an artifact whereby the populations of some water scarce countries like Egypt and Saudi Arabia [41] had little or no drought exposure during the period of analysis. Countries with little to no rainfall may be highly vulnerable to small decreases in that rainfall. Therefore, future work should incorporate a term for water scarcity that will attempt to quantify this difference in vulnerability.

Drought exposure was greater as HDI class decreased. Very high HDI countries had 64% less exposure compared to the global mean and low HDI countries had 33% greater exposure compared to the global mean (see Table 3).

### 3.3. Flood Country-level Population Exposure

**Figure 6:**
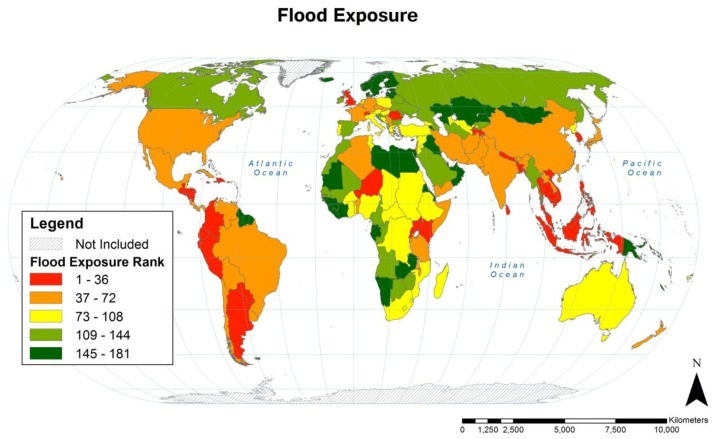
Rankings of country-level population flood exposure expressed as quintiles. Forty-eight countries (of 228) had no floods in populated areas during the 1985–2003 period of analysis and are each tied for 181st rank.

Country-Level rankings of population exposure to flood are illustrated in Figure 6 and the rankings of the countries with the greatest population flood exposure are listed in Table 3.

Like drought, flood exposure is more widely distributed geographically than cyclones. Populations of nearly all Southeast Asian countries were highly exposed to flooding during the 1985–2003 period of analysis and likewise for most of the Americas (including the Caribbean), South Asia, and East Asia. In contrast to cyclone and drought, flood exposure was moderate to high for the populations of most European countries. Flood exposure by HDI class was highest in medium HDI countries with exposure 12% above the global mean. The other three HDI classes had lower exposure than the global mean, with high HDI countries the lowest at 22% below the average (see Table 3).

### 3.4. Total Country-level Population Exposure: Multi-hazard

**Figure 7:**
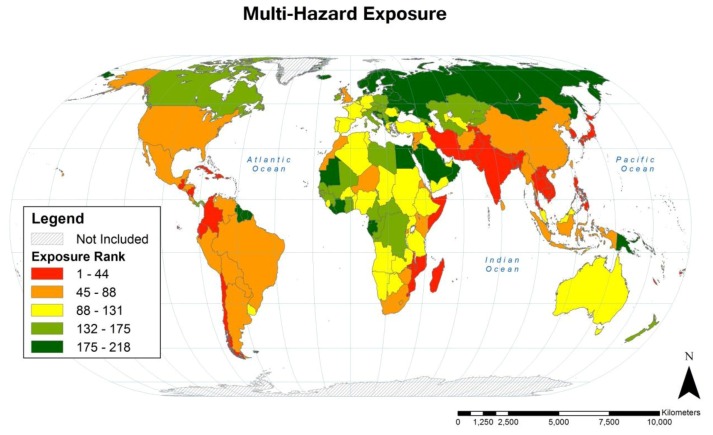
Rankings of country-level population multi-hazard (cyclone, drought, and flood) exposure over the 1980–2000 period of analysis, expressed as quintiles.

Summing the population exposure scores for cyclone, drought and flood yields a total multi-hazard population exposure score. These are illustrated as country rankings in quintiles in Figure 7. 

**Table 5:** Ranked lists of the twenty countries with the greatest multi-hazard population exposure with country rankings for cyclone, drought, and flood ^1^.

Country	Cyclone Rank	Drought Rank	Flood Rank	Multi-Hazard Exposure Rank
Hong Kong	5	139	3	1
Philippines	11	74	22	2
Macao	10	132	1	3
Guatemala	63	10	5	4
South Korea	22	118	15	5
Bangladesh	53	29	2	6
Vietnam	36	80	12	7
Saint Kitts and Nevis	20	6	181	8
Guadeloupe	17	65	83	9
Guam	1	68	132	10
Lebanon	93	2	42	11
Ecuador	93	27	17	12
Nepal	93	44	6	13
Japan	7	182	64	14
British Virgin Islands	8	45	181	15
Thailand	73	35	20	16
Puerto Rico	14	193	48	17
Antigua and Barbuda	9	70	134	18
New Caledonia	6	66	166	19
Mozambique	40	31	73	20

Note: ^1^ The complete list of countries and rankings is included in the Online Supplementary Information.

Additionally, the countries with the greatest population multi-hazard exposure and their rankings for cyclone, drought and flood are included in Table 5. Multi-hazard exposure by HDI class was highest in medium HDI countries with exposure 17% greater than the global mean and lowest in high HDI countries with 32% less exposure than the global mean (see Table 3).

The multi-hazard population exposure score provides an interesting summary of exposure to these three climate-related hazards. However, exposure to cyclone, drought and flood should also be evaluated separately, as appropriate to the outcome or the adaptation measure being studied. For example, reducing vulnerability to drought-related impacts on agriculture will, in practice, be different to mitigating flood impacts on drinking water quality and sanitation.

### 3.5. Urban/Rural Population Exposure

Urban and rural population exposure scores can be used to compare the relative population exposure of urban and rural dwellers within and between countries. In most countries, urban and rural populations differ systematically across numerous socio-economic and other resilience parameters. Thus identifying exposure to specific hazards informs likely impacts and the vulnerability of urban and rural populations. Although differences in urban and rural resilience and adaptive capacity are often discussed, this analysis focuses on differences in the exposure of urban and rural populations.

Comparing average global exposure of rural to urban populations revealed that exposure in urban populations was 40% higher for cyclones than rural populations and 34% lower for droughts. There was no difference for floods. Although the global trend for flood exposure indicates no trend and no systematic difference in flood exposure, country-level associations are present for cyclones, droughts, and floods. This may be a result of geographic location of urban and rural areas (e.g., urban areas on the coast are affected by cyclones) as well as the many different causes of flood (e.g., due to snowmelt, avalanche, storm surge, heavy precipitation). The differences between urban and rural exposure within HDI class were greatest for cyclones in very high and medium HDI countries: average urban cyclone exposure was greater than rural by 41% in very high HDI countries and 47% for medium HDI countries. Average exposure to drought was greater in urban than rural populations for very high HDI countries by 21% and 11% for low HDI countries but greater in rural than in urban populations by 12% for medium HDI countries. Average exposure to flood was greater in urban than rural populations by 14% and 12% for very high and high HDI countries. Low and medium HDI countries have less than 10% difference between urban and rural exposure to flood. See Table 3 for all average exposure values for rural and urban populations compared to the average global and average hazard exposure.

Although urban and rural population exposure may be used to compare countries, these values may also be used to compare urban and rural populations within a country. In a number of countries, including Brazil, Ghana and Turkey, flood exposure of urban populations was much greater than that of rural populations, while drought exposure was greater for rural populations. Alternatively, a number of countries have rural or urban populations consistently more exposed across all hazards. In Chile, urban exposure was higher than rural exposure for both drought and flood, with the opposite seen in Costa Rica. Full tables of urban and rural exposure values for each country and each hazard are included in the Online Supplementary Information (see Tables S7–S11).

### 3.6. Limitations

The limitations of any model are dependent on the quality of the data used. This model was built with the goal to enable incorporation of new datasets. Although the best available global datasets were used, the limitations of these datasets and their integration into the model are described below, starting with population, then urban extent and finally hazard event datasets.

The global population datasets we compared are all dependent on national census data. National census collection methods vary in administrative unit size and data collection frequencies. Although we determined that LandScan^TM^ was the best global population density dataset for this assessment, other datasets may be better suited for analyses focusing on a given country or continent. For example, Afripop [42] is a freely available, fully documented, high resolution population density dataset for Africa (0.05' × 0.05', ~100 m) that was created using the most recent census data supplemented with satellite imagery for land use and settlements. The impacts of using different global population datasets on analysis results have been evaluated by others; dataset choice had little impact at global scale but larger impacts on sub-national scales [21].

Urban extent is defined in our analysis using a country-specific population density threshold. The reliability of identifying urban extent using a population density threshold is dependent on the spatial resolution of the population density data [21,43]. Definitions of urban and rural areas differ. In fact the spatial distribution of urban areas consists of a spectrum rather than binary urban or rural classes. We recognize that a binary definition of urban and rural does not necessarily capture the gradual changes between a city and agricultural area with a peri-urban perimeter or potentially suburban areas. Additionally, uninhabited areas are conflated with low density areas and both identified as rural. Despite these shortcomings, identifying urban areas is useful for assessing the potential resilience and adaptive capacity of a setting to a broad variety of climate-related impacts. Using a population density approach to define urban and rural areas allows a consistent application globally without imposing a uniform population density threshold. Most importantly, it allows the application of our model to a broad range of climate-related outcomes and adaptation measures; many other methods to define urban extent use parameters such as infrastructure, which can result in double-counting of input variables and outcomes (*i.e.*, the input variable and the outcome are not independent of each other).

CHRR’s relative hazard frequency data is limited primarily by the twenty-year recording period, potential differences in hazard reporting between countries and urban/rural settings, and the spatial resolution of the data. Hazard frequency data are dependent on the approximately twenty-year time frame for which reported hazards were identified. Due to the short period of the dataset, hazard reporting for rare, extreme events is limited [4]. For example, the Republic of the Marshall Islands had no exposure to drought or flood over the period of analysis, but had both severe drought and flood events in 2013 [44,45]. As climate changes, hazard frequencies will also change over time. These historic hazard relative frequency data inform estimates of prevalent exposure rather than predicting future likelihood of hazard occurrence.

Additionally, CHRR data are dependent on the reported hazards. Using data based on reporting of hazard events may result in under-estimation of hazards from developing countries or more rural areas [4] and also misclassification of hazards since hazard reporting is not standardized globally. It is assumed more likely that larger events are identified and recorded more often than smaller events (e.g., major flooding versus flash floods) [4]. Frequency of hazard event should also clearly identify what duration of a hazard event constitutes separate events. A longer time frame of analysis as well as consistency in reporting in all settings would be preferable.

Finer resolution datasets would be preferable; however we have used CHRR relative hazard frequency data, the best global data available. Using hazard datasets with finer spatial resolution would be beneficial to ensure that the model’s exposure results validate the assumption that there is homogeneity over the scale at which the hazard data are recorded. If homogeneity over the spatial scale does not hold true, urban or rural areas with a much finer resolution will be weighted similarly when, in fact, they are affected differently by the hazard, although we would not be able to say whether a systematic difference occurs between urban and rural areas. Future work should incorporate data with higher resolution hazard data to validate whether differences in urban and rural exposure hold true at different spatial scales. The scale of cyclone data is comparable to the scale of the population data, but drought and flood data are much coarser resolution than population data. For drought, this is probably due to the scale of actual drought events affecting larger areas. For floods, CHRR models extreme flood events over large areas rather than localized flooding events (e.g., flash floods). This exposure model is built to incorporate new data and is built so hazard data with higher resolution can be included when they become available.

Including hazard severity would be desirable. However, the current state of the science does not justify the comparison of hazard severity between hazard types and hazard severity may have to be weighted differently depending on the outcome(s) being studied [4]. CHRR indicates that “globally uniform data do not yet exist to produce a justifiable measure of hazard severity that can be applied consistently across multiple hazards” [4]. More research is needed to develop a multi-hazard approach to normalizing exposure and likelihood of hazard event. Therefore, a binary measure of the relative frequency of hazard events was considered most appropriate for this multi-hazard analysis.

Although it would be preferable to project the probability of future hazard events, we model relative likelihood of historic hazards rather than a predictive model of historic or future events because GCC models using historic likelihood data are not adequate for accurately projecting the frequency of future hazard events. The relative hazard frequency datasets that we used are limited by incomplete hazard occurrence reporting over a twenty year period and variations in reporting frequency; preferably a reliable longer-term record of hazard events would be available, although a longer term record, even if available, could average hazard occurrences rather than capture changes in climate-related hazard events over time.

Drought modeled by CHRR does not incorporate a measure for aridity. The impact of a drought (defined in the CHRR dataset as precipitation less than 50% of average over three consecutive months) on the outcome being modeled is likely to depend on aridity.

## 4. Conclusions

We analyzed exposure to the climate-related hazard events cyclone, drought and flood, and a multi-hazard parameter summing total exposure over these three hazard events. We focused on population exposure (relative hazard frequency in a given area weighted by population density). This approach enables the application of population exposure to diverse climate-related outcomes (e.g., mortality due to hazards, as previous studies have done, or population at risk to loss of drinking water or agricultural productivity).

Our model is designed to be easily updated and refined as data become available. Our analysis displays relative exposure of urban and rural populations independent of variables associated with resilience and adaptive capacity. We distinguish between urban and rural exposure to allow different parameters to be applied to these areas (e.g., socioeconomic factors, access to health care or water utilities) in later vulnerability assessments of any kind. These exposure estimates are independent of the susceptibility or adaptive capacity of a particular system.

The geographic distribution of average population exposure varied for the three hazard events. Cyclone exposure was highly concentrated in five geographic regions, with over half of countries experiencing no cyclones in populated areas during the period of analysis. Drought exposure was more broadly distributed than cyclone exposure, with populations of countries in South Asia, Southeast Asia and Western Asia through the Mediterranean most highly exposed. Flood exposure was also more widely distributed than cyclone exposure, with Southeast Asian countries again highly exposed; exposure was also high in the Americas, South Asia, East Asia, and through much of Europe. Globally, urban populations were more exposed to cyclone and rural populations more exposed to drought; there was little difference in urban and rural exposure to flood.

Average exposure was compared across countries in the four HDI categories. Cyclone exposure was greatest in very high HDI countries. In contrast, drought disproportionately affected low and medium HDI countries. Flood exposure was highest in medium HDI countries but was more evenly distributed across HDI categories than were cyclone and drought. These analyses were also carried out for urban and rural areas by HDI. Exposure to cyclones was greatest in very high HDI countries and higher for urban than rural populations, whereas exposure to drought was greatest in low HDI countries for both urban and rural populations.

This exposure assessment has implications for country-level adaptation. It could be used to help prioritize aid decisions and allocation of adaptation resources within a country. Additionally this assessment compares exposure to a particular hazard across countries, allowing comparison between the total, urban and rural populations of different countries. This model is designed to allow flexibility in applying exposure assessment to a range of outcomes and adaptation measures, for example mortality, economic loss, water infrastructure repair costs, sanitation risks, or other outcomes due to these climate-related hazards.

Future research should continue to refine how hazard severity and intensity can be integrated into hazard frequency datasets through probabilistic or stochastic means and used to inform risk assessments. As new research continues to identify urban and rural areas incorporating satellite imagery with population density measurements, we hope that a population density approach allows a consistent application of globally available data as a base measure of urbanity that will also allow for degrees of urban-ness to be identified. As these new and better datasets become available, model outputs will be updated and made available on the Water Institute website [39]. Additionally, incorporating sea level rise, water scarcity and aridity, and other climate-related factors will enable application of this model to projecting the impacts of climate change.

## Supplementary Files

Supplementary File 1 Supplementary Information (PDF, 319 KB)
