# Supplementary material for: Climate-Related Hazards: A Method for Global Assessment of Urban and Rural Population Exposure to Cyclones, Droughts, and Floods
Source: Int J Environ Res Public Health. 2014 Feb 21;11(2):2169–92. doi: 10.3390/ijerph110202169 (PMC3945591; doi:10.3390/ijerph110202169)
Supplement: Supplementary File 1 — Supplementary Information (PDF, 319 KB) [file ijerph-11-02169-s001.pdf]

Climate-Related Hazards: A Method for Global Assessment of Urban and Rural Population Exposure to Cyclone, Drought, and Flood

ArcGIS Toolbox: Exposure Assessment for Global Model [1]

Table S1. Comparison of previous global hazard risk assessments.

Assessment	DRI	GNDRH	GAR 2009	GAR 2013
Citation	UNDP 2004	Dilley *et al*. 2005 [2]	UNISDR, *Global Assessment Report on Disaster Risk Reduction*. 2009 [3]	UNISDR*, Global Assessment Report on Disaster Risk Reduction*. 2013 [4]
Input Data	Hazard: frequency of cyclone, drought, flood, earthquake Observed Mortality: EM-DAT	Hazard: cyclone, drought, earthquake, flood, landslide, volcano Observed Mortality: EM-DAT	Hazard: cyclone, drought, earthquake, flood, landslide Observed Mortality and Economic Loss: EM-DAT	Hazard: cyclone, earthquake
Vulnerability Variables	rate of urban growth, gross domestic product per capita, percent arable land, human development index, percent population with access to improved water supply	region of the world, wealth status	government accountability, per capita income, remoteness, urban growth	building class: income, construction quality, education hazard measurement parameter: varies depending on hazard (e.g., water depth for flooding) identifies variables that best explain mortality risk to different types of hazards
Outcomes Modelled	relative risk of hazard mortality	relative risk of hazard mortality, relative risk of economic loss due to hazard	relative risk of hazard mortality, relative risk of economic loss due to hazard	urban economic loss expressed as likelihood per year

Table S1. *Cont.*

Assessment	DRI	GNDRH	GAR 2009	GAR 2013
Estimation Method	multiplied population exposed (at time of event) by hazard frequency and model-selected vulnerability variables to predict observed mortality for a hazard using multiple regression equation for each hazard	multiplied current population exposed by hazard frequency weighted by vulnerability coefficients at each grid cell and allocated mortality proportionally to equal observed mortality	multiplied current population by hazard frequency weighted by vulnerability coefficients at each grid cell and allocated mortality proportionally to equal observed mortality	each urban grid cell is given a building class. Probabilistic model statistically represents frequency of future hazard events. Vulnerability is determined by modeling relationship between building class and measurement parameter of the hazard

Table S2. Population exposure rank by country.

Country	Rural Per Capita Multi-Hazard Exposure Rank	Urban Per Capita Multi-Hazard Exposure Rank	Cyclone Rank	Drought Rank	Flood Rank	Multi-Hazard Exposure Rank
Afghanistan	49	16	93	32	45	46
Albania	172	188	93	178	114	186
Algeria	94	89	93	83	71	89
American Samoa	179	185	62	136	181	189
Andorra	178	179	93	141	127	185
Angola	107	81	93	23	125	93
Anguilla	N/A	30	16	45	151	22
Antigua and Barbuda	13	26	9	70	134	18
Argentina	95	66	93	92	36	67
Armenia	165	202	93	137	156	195
Aruba	166	182	75	142	131	179
Australia [1]	133	100	71	95	74	106
Austria	149	152	93	193	75	154
Azerbaijan	122	131	93	50	140	130
Bahamas	99	134	29	181	181	129
Bahrain	205	214	93	193	178	217
Bangladesh	3	8	53	29	2	6
Barbados	102	105	58	45	133	111
Belarus	171	189	93	193	115	190
Belgium	112	133	93	172	53	131
Belize	44	44	24	193	76	48
Benin	79	108	93	90	67	99

Table S2. *Cont.*

Country	Rural Per Capita Multi-Hazard Exposure Rank	Urban Per Capita Multi-Hazard Exposure Rank	Cyclone Rank	Drought Rank	Flood Rank	Multi-Hazard Exposure Rank
Bermuda	N/A	74	21	193	127	78
Bhutan	31	47	93	21	56	41
Bolivia	77	75	93	101	39	81
Bosnia and Herzegovina	161	171	93	170	98	171
Botswana	117	126	93	43	138	125
Brazil	101	83	93	98	46	87
British Virgin Islands	16	20	8	45	181	15
Brunei	187	199	93	131	172	200
Bulgaria	183	197	93	153	144	198
Burkina Faso	115	111	93	89	94	121
Burundi	80	116	93	106	47	91
Cambodia	26	54	86	84	14	37
Cameroon	163	158	93	119	111	165
Canada	160	169	59	124	139	168
Cape Verde	138	160	77	76	181	155
Cayman Islands	N/A	137	32	184	181	135
Central African Republic	144	128	93	116	90	145
Chad	104	123	93	61	105	114
Channel Islands [2]	191	184	70	146	181	197
Chile	81	21	93	18	37	24
China	54	49	49	87	49	52
Colombia	37	35	88	55	16	34
Comoros [3]	123	146	39	126	152	134
Congo, Republic of the	154	155	93	113	110	159
Cook Islands	143	156	45	112	181	156
Costa Rica	58	93	60	107	44	79
Côte d'Ivoire	176	198	93	125	155	191
Croatia	182	194	93	174	123	194
Cuba	17	41	35	97	60	36
Cyprus	128	147	93	51	181	144
Czech Republic	131	135	93	193	57	137

Table S2. *Cont.*

Country	Rural Per Capita Multi-Hazard Exposure Rank	Urban Per Capita Multi-Hazard Exposure Rank	Cyclone Rank	Drought Rank	Flood Rank	Multi-Hazard Exposure Rank
Democratic Republic of the Congo	157	150	93	121	108	162
Denmark	185	201	93	145	181	202
Djibouti	64	69	93	7	150	70
Dominica	24	38	19	70	135	33
Dominican Republic	12	31	30	154	24	21
Ecuador	14	12	93	27	17	12
Egypt	199	204	93	193	153	207
El Salvador	66	70	85	183	9	71
Equatorial Guinea	197	196	93	147	181	203
Eritrea	71	85	93	13	149	83
Estonia	202	210	93	187	181	211
Ethiopia	87	76	93	36	107	90
Faeroe Islands	208	214	93	193	181	218
Falkland Islands	206	214	93	192	181	216
Fiji	30	28	23	100	89	30
Finland [4]	198	209	93	179	181	209
France	132	127	80	160	54	128
French Guiana	208	214	93	193	181	218
French Polynesia	146	119	34	163	181	136
Gabon	184	193	93	143	157	196
Gambia	140	154	93	63	181	153
Georgia	150	157	93	171	82	157
Germany	111	115	93	155	43	116
Ghana	152	159	93	127	100	161
Gibraltar	N/A	61	93	1	181	62
Greece	110	82	93	38	106	98
Greenland	200	206	93	158	181	206
Grenada	181	162	44	193	181	178
Guadeloupe [5]	7	10	17	65	83	9
Guam	8	14	1	68	132	10
Guatemala	2	4	63	10	5	4
Guinea	162	138	93	85	163	160
Guinea-Bissau	158	174	93	96	181	173

Table S2. *Cont.*

Country	Rural Per Capita Multi-Hazard Exposure Rank	Urban Per Capita Multi-Hazard Exposure Rank	Cyclone Rank	Drought Rank	Flood Rank	Multi-Hazard Exposure Rank
Guyana	174	176	93	111	162	180
Haiti	32	23	38	151	13	26
Honduras	52	55	56	148	10	55
Hong Kong [6]	N/A	2	5	139	3	1
Hungary	145	165	93	168	85	163
Iceland	208	214	93	193	181	218
India	27	29	68	25	41	31
Indonesia	51	45	83	60	27	50
Iran	38	43	93	24	50	43
Iraq	82	96	93	17	146	96
Ireland	180	167	64	185	113	174
Isle of Man	208	214	93	193	181	218
Israel	61	97	93	86	70	92
Italy	141	151	93	144	84	152
Jamaica	39	40	48	166	4	44
Japan	29	19	7	182	64	14
Jordan	40	48	93	8	95	49
Kazakhstan	139	143	93	58	154	148
Kenya	46	50	93	91	18	51
Kiribati	97	107	93	19	181	108
Kuwait	194	212	93	188	175	212
Kyrgyzstan	126	130	93	53	137	133
Laos	21	37	42	39	55	29
Latvia	207	213	93	191	181	215
Lebanon	11	13	93	2	42	11
Lesotho	48	80	93	16	99	57
Liberia	190	186	93	123	170	193
Libya	142	149	93	57	180	150
Liechtenstein	65	98	93	193	7	73
Lithuania	201	208	93	193	165	210
Luxembourg	86	94	93	186	25	97
Macao [6]	N/A	3	10	132	1	3
Macedonia	135	148	93	173	66	149
Madagascar	22	17	12	102	96	23
Malawi	34	36	61	20	68	42
Malaysia	103	103	93	161	32	109
Maldives	208	214	93	193	181	218
Mali	134	136	93	81	122	142
Malta	67	71	93	2	181	73
Marshall Islands	208	214	93	193	181	218
Martinique	147	191	51	193	160	188

Table S2. *Cont.*

Country	Rural Per Capita Multi-Hazard Exposure Rank	Urban Per Capita Multi-Hazard Exposure Rank	Cyclone Rank	Drought Rank	Flood Rank	Multi-Hazard Exposure Rank
Mauritania	177	178	93	115	158	184
Mauritius	56	60	4	169	181	61
Mexico	41	46	57	40	52	47
Micronesia, Federated States of	55	56	28	67	181	58
Moldova, Republic of	159	170	93	122	117	169
Monaco	N/A	141	93	132	77	140
Mongolia	192	163	93	99	169	175
Montenegro	169	173	93	193	104	176
Montserrat	45	5	15	68	181	38
Morocco [7]	83	77	93	22	118	84
Mozambique	19	22	40	31	73	20
Myanmar	62	65	72	9	136	65
Namibia	124	120	93	33	179	127
Nauru	N/A	72	93	2	181	73
Nepal	10	11	93	44	6	13
Netherlands	151	164	93	150	97	166
Netherlands Antilles	119	118	46	59	168	117
New Caledonia	18	25	6	66	166	19
New Zealand	156	139	74	175	65	143
Nicaragua	36	18	52	49	29	27
Niger	50	52	93	75	28	53
Nigeria	92	91	93	54	86	94
Niue	89	117	33	109	181	107
North Korea [8]	47	59	37	105	79	56
Northern Mariana Islands	23	42	2	104	181	40
Norway	195	203	93	149	167	205
Oman	196	207	93	177	164	208
Pakistan	33	32	76	14	51	28
Palau	125	142	55	72	181	138
Palestine	35	86	93	37	80	68
Panama	137	145	87	156	72	147
Papua New Guinea	168	181	84	117	159	183
Paraguay	68	67	93	138	21	72
Peru	73	88	93	114	35	86

Table S2. *Cont.*

Country	Rural Per Capita Multi-Hazard Exposure Rank	Urban Per Capita Multi-Hazard Exposure Rank	Cyclone Rank	Drought Rank	Flood Rank	Multi-Hazard Exposure Rank
Philippines	1	1	11	74	22	2
Pitcairn Islands	208	214	93	193	181	218
Poland	153	161	93	162	88	164
Portugal	91	101	82	79	81	102
Puerto Rico	15	24	14	193	48	17
Qatar	204	211	93	193	176	214
Réunion	60	62	3	193	181	63
Romania	98	109	93	159	33	110
Russia	167	175	78	130	126	177
Rwanda	74	90	93	180	19	85
Saint Helena	208	214	93	193	181	218
Saint Kitts and Nevis	6	9	20	6	181	8
Saint Lucia	175	183	54	193	147	181
Saint Pierre and Miquelon	121	104	26	193	181	112
Saint Vincent and the Grenadines	170	153	41	193	181	167
Samoa	148	166	47	140	142	158
San Marino	189	200	93	193	127	201
Sao Tome and Principe	208	214	93	193	181	218
Saudi Arabia	186	190	93	167	121	192
Senegal	130	140	93	62	141	139
Serbia	127	132	93	176	58	132
Seychelles	208	214	93	193	181	218
Sierra Leone	118	106	93	34	148	119
Singapore	N/A	72	93	193	7	73
Slovakia	129	144	93	193	63	141
Slovenia	193	205	93	193	143	204
Solomon Islands	155	192	50	152	145	170
Somalia	42	15	93	15	62	35
South Africa	59	78	91	42	78	69
South Korea [9]	5	6	22	118	15	5
Spain	108	124	89	64	109	122
Sri Lanka	53	79	67	135	11	59
Sudan [10]	90	87	93	52	91	95
Suriname	203	214	93	190	177	213
Swaziland	28	53	81	2	87	39

Table S2. *Cont.*

Country	Rural Per Capita Multi-Hazard Exposure Rank	Urban Per Capita Multi-Hazard Exposure Rank	Cyclone Rank	Drought Rank	Flood Rank	Multi-Hazard Exposure Rank
Sweden	188	195	93	128	173	199
Switzerland	88	99	93	193	26	101
Syria	57	51	93	11	102	54
Tajikistan	25	33	93	28	31	32
Tanzania, United Republic of	76	84	93	78	69	88
Thailand	9	39	73	35	20	16
Timor-Leste	84	112	69	41	112	100
Togo	120	168	93	103	103	146
Tokelau	208	N/A	93	193	181	218
Tonga	75	64	31	88	181	80
Trinidad and Tobago	106	121	79	193	34	113
Tunisia	109	129	93	93	92	126
Turkey	116	114	93	77	101	118
Turkmenistan	164	172	93	120	120	172
Turks and Caicos Islands	85	102	25	193	181	104
Tuvalu	105	180	66	82	181	151
Uganda	96	95	93	157	30	105
Ukraine	173	187	93	165	119	187
United Arab Emirates	136	110	93	26	174	115
United Kingdom	78	68	65	164	23	77
United States of America	69	63	43	108	59	66
United States Virgin Islands	20	34	13	48	171	25
Uruguay	93	125	93	129	61	123
Uzbekistan	100	92	93	56	93	103
Vanuatu	43	27	18	94	124	45
Vatican City State [11]	N/A	177	93	132	127	182
Venezuela	72	57	90	73	38	60
Vietnam	4	7	36	80	12	7
Wallis and Futuna Islands	70	N/A	27	110	181	82

Table S2. *Cont.*

Country	Rural Per Capita Multi-Hazard Exposure Rank	Urban Per Capita Multi-Hazard Exposure Rank	Cyclone Rank	Drought Rank	Flood Rank	Multi-Hazard Exposure Rank
Yemen	114	122	93	189	40	124
Zambia	113	113	93	30	161	120
Zimbabwe	63	58	92	12	116	64

Notes: [1] includes Christmas Island and Cocos islands; [2] includes Guernsey and Jersey; [3] includes Mayotte; [4] includes Aland Islands; [5] includes Saint Barthelemy and Saint Martin; [6] Special Administrative Region (SAR) of China; [7] includes Western Sahara; [8] Democratic People's Republic of Korea; [9] Republic of Korea; [10] includes Southern Sudan; [11] Holy See.

Table S3. Multi-hazard exposure and rank.

Country	Cyclone Rank	Drought Rank	Flood Rank	Multi-Hazard Total Exposure	Multi-Hazard Total Rank
Hong Kong [1]	5	139	3	2.085	1
Philippines	11	74	22	2.068	2
Macao [1]	10	132	1	1.995	3
Guatemala	63	10	5	1.872	4
South Korea [2]	22	118	15	1.807	5
Bangladesh	53	29	2	1.779	6
Vietnam	36	80	12	1.735	7
Saint Kitts and Nevis	20	6	181	1.700	8
Guadeloupe [3]	17	65	83	1.591	9
Guam	1	68	132	1.500	10
Lebanon	93	2	42	1.500	11
Ecuador	93	27	17	1.487	12
Nepal	93	44	6	1.470	13
Japan	7	182	64	1.430	14
British Virgin Islands	8	45	181	1.413	15
Thailand	73	35	20	1.401	16
Puerto Rico	14	193	48	1.400	17
Antigua and Barbuda	9	70	134	1.399	18
New Caledonia	6	66	166	1.389	19
Mozambique	40	31	73	1.388	20
Dominican Republic	30	154	24	1.388	21
Anguilla	16	45	151	1.376	22
Madagascar	12	102	96	1.372	23
Chile	93	18	37	1.352	24
United States Virgin Islands	13	48	171	1.350	25
Haiti	38	151	13	1.335	26
Nicaragua	52	49	29	1.333	27
Pakistan	76	14	51	1.330	28
Laos	42	39	55	1.328	29

Table S3. *Cont.*

Country	Cyclone Rank	Drought Rank	Flood Rank	Multi-Hazard Total Exposure	Multi-Hazard Total Rank
Fiji	23	100	89	1.323	30
India	68	25	41	1.310	31
Tajikistan	93	28	31	1.308	32
Dominica	19	70	135	1.296	33
Colombia	88	55	16	1.295	34
Somalia	93	15	62	1.272	35
Cuba	35	97	60	1.266	36
Cambodia	86	84	14	1.244	37
Montserrat	15	68	181	1.239	38
Swaziland	81	2	87	1.230	39
Northern Mariana Islands	2	104	181	1.229	40
Bhutan	93	21	56	1.228	41
Malawi	61	20	68	1.221	42
Iran	93	24	50	1.205	43
Jamaica	48	166	4	1.198	44
Vanuatu	18	94	124	1.193	45
Afghanistan	93	32	45	1.178	46
Mexico	57	40	52	1.169	47
Belize	24	193	76	1.161	48
Jordan	93	8	95	1.155	49
Indonesia	83	60	27	1.132	50
Kenya	93	91	18	1.127	51
China	49	87	49	1.097	52
Niger	93	75	28	1.084	53
Syria	93	11	102	1.081	54
Honduras	56	148	10	1.076	55
North Korea [4]	37	105	79	1.048	56
Lesotho	93	16	99	1.036	57
Micronesia, Federated States of	28	67	181	1.036	58
Sri Lanka	67	135	11	1.031	59
Venezuela	90	73	38	1.024	60
Mauritius	4	169	181	1.017	61
Gibraltar	93	1	181	1.000	62
Réunion	3	193	181	0.995	63
Zimbabwe	92	12	116	0.984	64
Myanmar	72	9	136	0.968	65
United States of America	43	108	59	0.964	66
Argentina	93	92	36	0.941	67
Palestine	93	37	80	0.919	68
South Africa	91	42	78	0.918	69
Djibouti	93	7	150	0.915	70
El Salvador	85	183	9	0.912	71
Paraguay	93	138	21	0.902	72

Table S3. *Cont.*

Country	Cyclone Rank	Drought Rank	Flood Rank	Multi-Hazard Total Exposure	Multi-Hazard Total Rank
Liechtenstein	93	193	7	0.900	73
Malta	93	2	181	0.900	73
Nauru	93	2	181	0.900	73
Singapore	93	193	7	0.900	73
United Kingdom	65	164	23	0.890	77
Bermuda	21	193	127	0.884	78
Costa Rica	60	107	44	0.879	79
Tonga	31	88	181	0.864	80
Bolivia	93	101	39	0.858	81
Wallis and Futuna Islands	27	110	181	0.847	82
Eritrea	93	13	149	0.837	83
Morocco [5]	93	22	118	0.834	84
Rwanda	93	180	19	0.830	85
Peru	93	114	35	0.825	86
Brazil	93	98	46	0.824	87
Tanzania, United Republic of	93	78	69	0.820	88
Algeria	93	83	71	0.794	89
Ethiopia	93	36	107	0.785	90
Burundi	93	106	47	0.783	91
Israel	93	86	70	0.776	92
Angola	93	23	125	0.775	93
Nigeria	93	54	86	0.774	94
Sudan [6]	93	52	91	0.772	95
Iraq	93	17	146	0.772	96
Luxembourg	93	186	25	0.770	97
Greece	93	38	106	0.764	98
Benin	93	90	67	0.754	99
Timor-Leste	69	41	112	0.753	100
Switzerland	93	193	26	0.751	101
Portugal	82	79	81	0.744	102
Uzbekistan	93	56	93	0.741	103
Turks and Caicos Islands	25	193	181	0.731	104
Uganda	93	157	30	0.730	105
Australia [7]	71	95	74	0.717	106
Niue	33	109	181	0.715	107
Kiribati	93	19	181	0.708	108
Malaysia	93	161	32	0.706	109
Romania	93	159	33	0.704	110
Barbados	58	45	133	0.699	111
Saint Pierre and Miquelon	26	193	181	0.699	112
Trinidad and Tobago	79	193	34	0.661	113
Chad	93	61	105	0.659	114
United Arab Emirates	93	26	174	0.653	115

Table S3. *Cont.*

Country	Cyclone Rank	Drought Rank	Flood Rank	Multi-Hazard Total Exposure	Multi-Hazard Total Rank
Germany	93	155	43	0.648	116
Netherlands Antilles	46	59	168	0.647	117
Turkey	93	77	101	0.643	118
Sierra Leone	93	34	148	0.638	119
Zambia	93	30	161	0.636	120
Burkina Faso	93	89	94	0.627	121
Spain	89	64	109	0.624	122
Uruguay	93	129	61	0.614	123
Yemen	93	189	40	0.612	124
Botswana	93	43	138	0.598	125
Tunisia	93	93	92	0.596	126
Namibia	93	33	179	0.592	127
France	80	160	54	0.579	128
Bahamas	29	181	181	0.574	129
Azerbaijan	93	50	140	0.563	130
Belgium	93	172	53	0.557	131
Serbia	93	176	58	0.540	132
Kyrgyzstan	93	53	137	0.539	133
Comoros [8]	39	126	152	0.538	134
Cayman Islands	32	184	181	0.530	135
French Polynesia	34	163	181	0.530	136
Czech Republic	93	193	57	0.528	137
Palau	55	72	181	0.519	138
Senegal	93	62	141	0.500	139
Monaco	93	132	77	0.500	140
Slovakia	93	193	63	0.493	141
Mali	93	81	122	0.493	142
New Zealand	74	175	65	0.486	143
Cyprus	93	51	181	0.485	144
Central African Republic	93	116	90	0.482	145
Togo	93	103	103	0.477	146
Panama	87	156	72	0.475	147
Kazakhstan	93	58	154	0.468	148
Macedonia	93	173	66	0.459	149
Libya	93	57	180	0.435	150
Tuvalu	66	82	181	0.428	151
Italy	93	144	84	0.428	152
Gambia	93	63	181	0.422	153
Austria	93	193	75	0.410	154
Cape Verde	77	76	181	0.402	155
Cook Islands	45	112	181	0.398	156
Georgia	93	171	82	0.378	157
Samoa	47	140	142	0.375	158

Table S3. *Cont.*

Country	Cyclone Rank	Drought Rank	Flood Rank	Multi-Hazard Total Exposure	Multi-Hazard Total Rank
Congo, Republic of the	93	113	110	0.375	159
Guinea	93	85	163	0.374	160
Ghana	93	127	100	0.372	161
Democratic Republic of the Congo	93	121	108	0.351	162
Hungary	93	168	85	0.350	163
Poland	93	162	88	0.348	164
Cameroon	93	119	111	0.337	165
Netherlands	93	150	97	0.333	166
Saint Vincent and the Grenadines	41	193	181	0.316	167
Canada	59	124	139	0.302	168
Moldova, Republic of	93	122	117	0.300	169
Solomon Islands	50	152	145	0.293	170
Bosnia and Herzegovina	93	170	98	0.293	171
Turkmenistan	93	120	120	0.270	172
Guinea-Bissau	93	96	181	0.267	173
Ireland	64	185	113	0.262	174
Mongolia	93	99	169	0.252	175
Montenegro	93	193	104	0.240	176
Russia	78	130	126	0.222	177
Grenada	44	193	181	0.220	178
Aruba	75	142	131	0.210	179
Guyana	93	111	162	0.210	180
Saint Lucia	54	193	147	0.203	181
Vatican City State [9]	93	132	127	0.200	182
Papua New Guinea	84	117	159	0.198	183
Mauritania	93	115	158	0.195	184
Andorra	93	141	127	0.193	185
Albania	93	178	114	0.187	186
Ukraine	93	165	119	0.179	187
Martinique	51	193	160	0.179	188
American Samoa	62	136	181	0.178	189
Belarus	93	193	115	0.173	190
Côte d'Ivoire	93	125	155	0.152	191
Saudi Arabia	93	167	121	0.147	192
Liberia	93	123	170	0.132	193
Croatia	93	174	123	0.130	194
Armenia	93	137	156	0.129	195
Gabon	93	143	157	0.122	196
Channel Islands [10]	70	146	181	0.121	197
Bulgaria	93	153	144	0.119	198
Sweden	93	128	173	0.114	199
Brunei	93	131	172	0.105	200
San Marino	93	193	127	0.100	201

Table S3. *Cont.*

Country	Cyclone Rank	Drought Rank	Flood Rank	Multi-Hazard Total Exposure	Multi-Hazard Total Rank
Denmark	93	145	181	0.085	202
Equatorial Guinea	93	147	181	0.068	203
Slovenia	93	193	143	0.067	204
Norway	93	149	167	0.063	205
Greenland	93	158	181	0.044	206
Egypt	93	193	153	0.037	207
Oman	93	177	164	0.026	208
Finland [11]	93	179	181	0.013	209
Lithuania	93	193	165	0.011	210
Estonia	93	187	181	0.001	211
Kuwait	93	188	175	0.001	212
Suriname	93	190	177	0.001	213
Qatar	93	193	176	0.000	214
Latvia	93	191	181	0.000	215
Falkland Islands	93	192	181	0.000	216
Bahrain	93	193	178	0.000	217
Faeroe Islands	93	193	181	0.000	218
French Guiana	93	193	181	0.000	218
Iceland	93	193	181	0.000	218
Isle of Man	93	193	181	0.000	218
Maldives	93	193	181	0.000	218
Marshall Islands	93	193	181	0.000	218
Pitcairn Islands	93	193	181	0.000	218
Saint Helena	93	193	181	0.000	218
Sao Tome and Principe	93	193	181	0.000	218
Seychelles	93	193	181	0.000	218
Tokelau	93	193	181	0.000	218

Notes: [1] Special Administrative Region (SAR) of China; [2] Republic of Korea; [3] includes Saint Barthelemy and Saint Martin; ; [4] Democratic People's Republic of Korea; [5] includes Western Sahara; [6] includes Southern Sudan; [7] includes Christmas Island and Cocos islands; [8] includes Mayotte; [9] Holy See; [10] includes Guernsey and Jersey; [11] includes Aland Islands.

Table S4. Cyclone exposure and rank.

Country	Cyclone Exposure	Cyclone Rank
Guam	1.000	1
Northern Mariana Islands	1.000	2
Réunion	0.995	3
Mauritius	0.993	4
Hong Kong [1]	0.993	5
New Caledonia	0.976	6
Japan	0.975	7
British Virgin Islands	0.913	8

Table S4. *Cont.*

Country	Cyclone Exposure	Cyclone Rank
Antigua and Barbuda	0.900	9
Macao [1]	0.895	10
Philippines	0.870	11
Madagascar	0.852	12
United States Virgin Islands	0.850	13
Puerto Rico	0.842	14
Montserrat	0.839	15
Anguilla	0.839	16
Guadeloupe [2]	0.829	17
Vanuatu	0.807	18
Dominica	0.800	19
Saint Kitts and Nevis	0.800	20
Bermuda	0.784	21
South Korea [3]	0.780	22
Fiji	0.763	23
Belize	0.756	24
Turks and Caicos Islands	0.731	25
Saint Pierre and Miquelon	0.699	26
Wallis and Futuna Islands	0.647	27
Micronesia, Federated States of	0.636	28
Bahamas	0.564	29
Dominican Republic	0.543	30
Tonga	0.526	31
Cayman Islands	0.526	32
Niue	0.515	33
French Polynesia	0.498	34
Cuba	0.490	35
Vietnam	0.478	36
North Korea [4]	0.444	37
Haiti	0.403	38
Comoros [5]	0.385	39
Mozambique	0.355	40
Saint Vincent and the Grenadines	0.316	41
Laos	0.267	42
United States of America	0.237	43
Grenada	0.220	44
Cook Islands	0.219	45
Netherlands Antilles	0.219	46
Samoa	0.215	47
Jamaica	0.204	48
China	0.202	49
Solomon Islands	0.180	50
Martinique	0.159	51
Nicaragua	0.156	52

Table S4. *Cont.*

Country	Cyclone Exposure	Cyclone Rank
Bangladesh	0.154	53
Saint Lucia	0.153	54
Palau	0.122	55
Honduras	0.114	56
Mexico	0.102	57
Barbados	0.100	58
Canada	0.099	59
Costa Rica	0.094	60
Malawi	0.081	61
American Samoa	0.080	62
Guatemala	0.076	63
Ireland	0.074	64
United Kingdom	0.062	65
Tuvalu	0.052	66
Sri Lanka	0.051	67
India	0.049	68
Timor-Leste	0.045	69
Channel Islands [6]	0.041	70
Australia [7]	0.032	71
Myanmar	0.031	72
Thailand	0.027	73
New Zealand	0.023	74
Aruba	0.020	75
Pakistan	0.012	76
Cape Verde	0.012	77
Russia	0.010	78
Trinidad and Tobago	0.008	79
France	0.008	80
Swaziland	0.007	81
Portugal	0.007	82
Indonesia	0.006	83
Papua New Guinea	0.005	84
El Salvador	0.005	85
Cambodia	0.005	86
Panama	0.002	87
Colombia	0.001	88
Spain	0.001	89
Venezuela	0.001	90
South Africa	0.000	91
Zimbabwe	0.000	92
Afghanistan	0.000	93
Albania	0.000	93
Algeria	0.000	93
Andorra	0.000	93

Table S4. *Cont.*

Country	Cyclone Exposure	Cyclone Rank
Angola	0.000	93
Argentina	0.000	93
Armenia	0.000	93
Austria	0.000	93
Azerbaijan	0.000	93
Bahrain	0.000	93
Belarus	0.000	93
Belgium	0.000	93
Benin	0.000	93
Bhutan	0.000	93
Bolivia	0.000	93
Bosnia and Herzegovina	0.000	93
Botswana	0.000	93
Brazil	0.000	93
Brunei	0.000	93
Bulgaria	0.000	93
Burkina Faso	0.000	93
Burundi	0.000	93
Cameroon	0.000	93
Central African Republic	0.000	93
Chad	0.000	93
Chile	0.000	93
Congo, Republic of the	0.000	93
Côte d'Ivoire	0.000	93
Croatia	0.000	93
Cyprus	0.000	93
Czech Republic	0.000	93
Democratic Republic of the Congo	0.000	93
Denmark	0.000	93
Djibouti	0.000	93
Ecuador	0.000	93
Egypt	0.000	93
Equatorial Guinea	0.000	93
Eritrea	0.000	93
Estonia	0.000	93
Ethiopia	0.000	93
Faeroe Islands	0.000	93
Falkland Islands	0.000	93
Finland [8]	0.000	93
French Guiana	0.000	93
Gabon	0.000	93
Gambia	0.000	93
Georgia	0.000	93
Germany	0.000	93

Table S4. *Cont.*

Country	Cyclone Exposure	Cyclone Rank
Ghana	0.000	93
Gibraltar	0.000	93
Greece	0.000	93
Greenland	0.000	93
Guinea	0.000	93
Guinea-Bissau	0.000	93
Guyana	0.000	93
Hungary	0.000	93
Iceland	0.000	93
Iran	0.000	93
Iraq	0.000	93
Isle of Man	0.000	93
Israel	0.000	93
Italy	0.000	93
Jordan	0.000	93
Kazakhstan	0.000	93
Kenya	0.000	93
Kiribati	0.000	93
Kuwait	0.000	93
Kyrgyzstan	0.000	93
Latvia	0.000	93
Lebanon	0.000	93
Lesotho	0.000	93
Liberia	0.000	93
Libya	0.000	93
Liechtenstein	0.000	93
Lithuania	0.000	93
Luxembourg	0.000	93
Macedonia	0.000	93
Malaysia	0.000	93
Maldives	0.000	93
Mali	0.000	93
Malta	0.000	93
Marshall Islands	0.000	93
Mauritania	0.000	93
Moldova, Republic of	0.000	93
Monaco	0.000	93
Mongolia	0.000	93
Montenegro	0.000	93
Morocco [9]	0.000	93
Namibia	0.000	93
Nauru	0.000	93
Nepal	0.000	93
Netherlands	0.000	93

Table S4. *Cont.*

Country	Cyclone Exposure	Cyclone Rank
Niger	0.000	93
Nigeria	0.000	93
Norway	0.000	93
Oman	0.000	93
Palestine	0.000	93
Paraguay	0.000	93
Peru	0.000	93
Pitcairn Islands	0.000	93
Poland	0.000	93
Qatar	0.000	93
Romania	0.000	93
Rwanda	0.000	93
Saint Helena	0.000	93
San Marino	0.000	93
Sao Tome and Principe	0.000	93
Saudi Arabia	0.000	93
Senegal	0.000	93
Serbia	0.000	93
Seychelles	0.000	93
Sierra Leone	0.000	93
Singapore	0.000	93
Slovakia	0.000	93
Slovenia	0.000	93
Somalia	0.000	93
Sudan [10]	0.000	93
Suriname	0.000	93
Sweden	0.000	93
Switzerland	0.000	93
Syria	0.000	93
Tajikistan	0.000	93
Tanzania, United Republic of	0.000	93
Togo	0.000	93
Tokelau	0.000	93
Tunisia	0.000	93
Turkey	0.000	93
Turkmenistan	0.000	93
Uganda	0.000	93
Ukraine	0.000	93
United Arab Emirates	0.000	93
Uruguay	0.000	93
Uzbekistan	0.000	93
Vatican City State [11]	0.000	93
Yemen	0.000	93
Zambia	0.000	93

Table S4. *Cont.*

Notes: [1] Special Administrative Region (SAR) of China; [2] includes Saint Barthelemy and Saint Martin; [3] Republic of Korea; [4] Democratic People's Republic of Korea; [5] includes Mayotte; [6] includes Guernsey and Jersey; [7] includes Christmas Island and Cocos islands; [8] includes Aland Islands; [9] includes Western Sahara; [10] includes Southern Sudan; [11] Holy See.

Table S5. Drought exposure and rank.

Country	Drought Exposure	Drought Rank
Gibraltar	1.000	1
Lebanon	0.900	2
Malta	0.900	2
Nauru	0.900	2
Swaziland	0.900	2
Saint Kitts and Nevis	0.900	6
Djibouti	0.877	7
Jordan	0.865	8
Myanmar	0.848	9
Guatemala	0.842	10
Syria	0.838	11
Zimbabwe	0.813	12
Eritrea	0.799	13
Pakistan	0.777	14
Somalia	0.775	15
Lesotho	0.773	16
Iraq	0.720	17
Chile	0.717	18
Kiribati	0.708	19
Malawi	0.702	20
Bhutan	0.698	21
Morocco [1]	0.683	22
Angola	0.667	23
Iran	0.663	24
India	0.653	25
United Arab Emirates	0.652	26
Ecuador	0.651	27
Tajikistan	0.628	28
Bangladesh	0.626	29
Zambia	0.617	30
Mozambique	0.615	31
Afghanistan	0.611	32
Namibia	0.592	33
Sierra Leone	0.589	34
Thailand	0.559	35
Ethiopia	0.555	36
Palestine	0.540	37
Greece	0.533	38

Table S5. *Cont.*

Country	Drought Exposure	Drought Rank
Laos	0.529	39
Mexico	0.528	40
Timor-Leste	0.520	41
South Africa	0.517	42
Botswana	0.517	43
Nepal	0.515	44
Anguilla	0.500	45
Barbados	0.500	45
British Virgin Islands	0.500	45
United States Virgin Islands	0.498	48
Nicaragua	0.495	49
Azerbaijan	0.486	50
Cyprus	0.485	51
Sudan [2]	0.464	52
Kyrgyzstan	0.452	53
Nigeria	0.451	54
Colombia	0.442	55
Uzbekistan	0.438	56
Libya	0.435	57
Kazakhstan	0.431	58
Netherlands Antilles	0.425	59
Indonesia	0.425	60
Chad	0.423	61
Senegal	0.423	62
Gambia	0.422	63
Spain	0.422	64
Guadeloupe [3]	0.408	65
New Caledonia	0.408	66
Micronesia, Federated States of	0.400	67
Guam	0.400	68
Montserrat	0.400	68
Antigua and Barbuda	0.400	70
Dominica	0.400	70
Palau	0.397	72
Venezuela	0.396	73
Philippines	0.393	74
Niger	0.392	75
Cape Verde	0.390	76
Turkey	0.388	77
Tanzania, United Republic of	0.385	78
Portugal	0.381	79
Vietnam	0.379	80
Mali	0.377	81
Tuvalu	0.375	82

Table S5. *Cont.*

Country	Drought Exposure	Drought Rank
Algeria	0.368	83
Cambodia	0.367	84
Guinea	0.359	85
Israel	0.346	86
China	0.343	87
Tonga	0.338	88
Burkina Faso	0.334	89
Benin	0.315	90
Kenya	0.303	91
Argentina	0.300	92
Tunisia	0.290	93
Vanuatu	0.277	94
Australia [4]	0.273	95
Guinea-Bissau	0.267	96
Cuba	0.264	97
Brazil	0.259	98
Mongolia	0.249	99
Fiji	0.249	100
Bolivia	0.238	101
Madagascar	0.238	102
Togo	0.236	103
Northern Mariana Islands	0.229	104
North Korea [5]	0.221	105
Burundi	0.219	106
Costa Rica	0.218	107
United States of America	0.209	108
Niue	0.200	109
Wallis and Futuna Islands	0.200	110
Guyana	0.193	111
Cook Islands	0.179	112
Congo, Republic of the	0.177	113
Peru	0.175	114
Mauritania	0.172	115
Central African Republic	0.171	116
Papua New Guinea	0.171	117
South Korea [6]	0.163	118
Cameroon	0.144	119
Turkmenistan	0.141	120
Democratic Republic of the Congo	0.138	121
Moldova, Republic of	0.137	122
Liberia	0.130	123
Canada	0.124	124
Côte d’Ivoire	0.118	125
Comoros [7]	0.116	126

Table S5. *Cont.*

Country	Drought Exposure	Drought Rank
Ghana	0.113	127
Sweden	0.113	128
Uruguay	0.108	129
Russia	0.107	130
Brunei	0.103	131
Macao [8]	0.100	132
Monaco	0.100	132
Vatican City State [9]	0.100	132
Sri Lanka	0.100	135
American Samoa	0.098	136
Armenia	0.095	137
Paraguay	0.095	138
Hong Kong [8]	0.095	139
Samoa	0.093	140
Andorra	0.093	141
Aruba	0.091	142
Gabon	0.089	143
Italy	0.087	144
Denmark	0.085	145
Channel Islands [10]	0.081	146
Equatorial Guinea	0.068	147
Honduras	0.067	148
Norway	0.059	149
Netherlands	0.058	150
Haiti	0.057	151
Solomon Islands	0.055	152
Bulgaria	0.054	153
Dominican Republic	0.052	154
Germany	0.051	155
Panama	0.050	156
Uganda	0.050	157
Greenland	0.044	158
Romania	0.044	159
France	0.039	160
Malaysia	0.034	161
Poland	0.034	162
French Polynesia	0.032	163
United Kingdom	0.031	164
Ukraine	0.029	165
Jamaica	0.027	166
Saudi Arabia	0.027	167
Hungary	0.026	168
Mauritius	0.024	169
Bosnia and Herzegovina	0.024	170

Table S5. *Cont.*

Country	Drought Exposure	Drought Rank
Georgia	0.022	171
Belgium	0.021	172
Macedonia	0.019	173
Croatia	0.018	174
New Zealand	0.017	175
Serbia	0.015	176
Oman	0.015	177
Albania	0.013	178
Finland [11]	0.013	179
Rwanda	0.012	180
Bahamas	0.010	181
Japan	0.009	182
El Salvador	0.008	183
Cayman Islands	0.005	184
Ireland	0.004	185
Luxembourg	0.003	186
Estonia	0.001	187
Kuwait	0.001	188
Yemen	0.000	189
Suriname	0.000	190
Latvia	0.000	191
Falkland Islands	0.000	192
Austria	0.000	193
Bahrain	0.000	193
Belarus	0.000	193
Belize	0.000	193
Bermuda	0.000	193
Czech Republic	0.000	193
Egypt	0.000	193
Faeroe Islands	0.000	193
French Guiana	0.000	193
Grenada	0.000	193
Iceland	0.000	193
Isle of Man	0.000	193
Liechtenstein	0.000	193
Lithuania	0.000	193
Maldives	0.000	193
Marshall Islands	0.000	193
Martinique	0.000	193
Montenegro	0.000	193
Pitcairn Islands	0.000	193
Puerto Rico	0.000	193
Qatar	0.000	193
Réunion	0.000	193

Table S5. *Cont.*

Country	Drought Exposure	Drought Rank
Saint Helena	0.000	193
Saint Lucia	0.000	193
Saint Pierre and Miquelon	0.000	193
Saint Vincent and the Grenadines	0.000	193
San Marino	0.000	193
Sao Tome and Principe	0.000	193
Seychelles	0.000	193
Singapore	0.000	193
Slovakia	0.000	193
Slovenia	0.000	193
Switzerland	0.000	193
Tokelau	0.000	193
Trinidad and Tobago	0.000	193
Turks and Caicos Islands	0.000	193

Notes: [1] includes Western Sahara; [2] includes Southern Sudan; [3] includes Saint Barthelemy and Saint Martin; [4] includes Christmas Island and Cocos islands; [5] Democratic People's Republic of Korea; [6] Republic of Korea; [7] includes Mayotte; [8] Special Administrative Region (SAR) of China; [9] Holy See; [10] includes Guernsey and Jersey; [11] includes Aland Islands.

Table S6. Flood exposure and rank.

Country	Flood Exposure	Flood Rank
Macao [1]	1.000	1
Bangladesh	0.999	2
Hong Kong [1]	0.997	3
Jamaica	0.966	4
Guatemala	0.954	5
Nepal	0.954	6
Liechtenstein	0.900	7
Singapore	0.900	7
El Salvador	0.899	9
Honduras	0.895	10
Sri Lanka	0.880	11
Vietnam	0.878	12
Haiti	0.874	13
Cambodia	0.872	14
South Korea [2]	0.864	15
Colombia	0.852	16
Ecuador	0.836	17
Kenya	0.823	18
Rwanda	0.818	19
Thailand	0.815	20
Paraguay	0.807	21
Philippines	0.805	22

Table S6. *Cont.*

Country	Flood Exposure	Flood Rank
United Kingdom	0.797	23
Dominican Republic	0.793	24
Luxembourg	0.768	25
Switzerland	0.751	26
Indonesia	0.701	27
Niger	0.692	28
Nicaragua	0.682	29
Uganda	0.680	30
Tajikistan	0.680	31
Malaysia	0.672	32
Romania	0.660	33
Trinidad and Tobago	0.653	34
Peru	0.650	35
Argentina	0.641	36
Chile	0.636	37
Venezuela	0.627	38
Bolivia	0.620	39
Yemen	0.612	40
India	0.608	41
Lebanon	0.600	42
Germany	0.596	43
Costa Rica	0.567	44
Afghanistan	0.567	45
Brazil	0.564	46
Burundi	0.564	47
Puerto Rico	0.557	48
China	0.552	49
Iran	0.543	50
Pakistan	0.541	51
Mexico	0.540	52
Belgium	0.536	53
France	0.532	54
Laos	0.531	55
Bhutan	0.529	56
Czech Republic	0.528	57
Serbia	0.525	58
United States of America	0.519	59
Cuba	0.511	60
Uruguay	0.506	61
Somalia	0.497	62
Slovakia	0.493	63
Japan	0.446	64
New Zealand	0.446	65
Macedonia	0.440	66

Table S6. *Cont.*

Country	Flood Exposure	Flood Rank
Benin	0.439	67
Malawi	0.438	68
Tanzania, United Republic of	0.435	69
Israel	0.431	70
Algeria	0.426	71
Panama	0.422	72
Mozambique	0.418	73
Australia [3]	0.412	74
Austria	0.410	75
Belize	0.406	76
Monaco	0.400	77
South Africa	0.400	78
North Korea [4]	0.382	79
Palestine	0.379	80
Portugal	0.357	81
Georgia	0.356	82
Guadeloupe [5]	0.355	83
Italy	0.341	84
Hungary	0.324	85
Nigeria	0.324	86
Swaziland	0.323	87
Poland	0.314	88
Fiji	0.311	89
Central African Republic	0.310	90
Sudan [6]	0.308	91
Tunisia	0.306	92
Uzbekistan	0.303	93
Burkina Faso	0.293	94
Jordan	0.290	95
Madagascar	0.281	96
Netherlands	0.275	97
Bosnia and Herzegovina	0.269	98
Lesotho	0.263	99
Ghana	0.258	100
Turkey	0.255	101
Syria	0.243	102
Togo	0.241	103
Montenegro	0.240	104
Chad	0.235	105
Greece	0.231	106
Ethiopia	0.230	107
Democratic Republic of the Congo	0.212	108
Spain	0.201	109
Congo, Republic of the	0.198	110

Table S6. *Cont.*

Country	Flood Exposure	Flood Rank
Cameroon	0.193	111
Timor-Leste	0.188	112
Ireland	0.183	113
Albania	0.175	114
Belarus	0.173	115
Zimbabwe	0.170	116
Moldova, Republic of	0.163	117
Morocco [7]	0.151	118
Ukraine	0.150	119
Turkmenistan	0.128	120
Saudi Arabia	0.120	121
Mali	0.116	122
Croatia	0.112	123
Vanuatu	0.110	124
Angola	0.108	125
Russia	0.105	126
Andorra	0.100	127
Bermuda	0.100	127
San Marino	0.100	127
Vatican City State [8]	0.100	127
Aruba	0.100	131
Guam	0.100	132
Barbados	0.099	133
Antigua and Barbuda	0.099	134
Dominica	0.096	135
Myanmar	0.088	136
Kyrgyzstan	0.087	137
Botswana	0.081	138
Canada	0.079	139
Azerbaijan	0.078	140
Senegal	0.077	141
Samoa	0.067	142
Slovenia	0.067	143
Bulgaria	0.065	144
Solomon Islands	0.058	145
Iraq	0.052	146
Saint Lucia	0.050	147
Sierra Leone	0.049	148
Eritrea	0.039	149
Djibouti	0.038	150
Anguilla	0.037	151
Comoros [9]	0.037	152
Egypt	0.037	153

Table S6. *Cont.*

Country	Flood Exposure	Flood Rank
Kazakhstan	0.036	154
Côte d'Ivoire	0.034	155
Armenia	0.034	156
Gabon	0.033	157
Mauritania	0.023	158
Papua New Guinea	0.022	159
Martinique	0.019	160
Zambia	0.019	161
Guyana	0.017	162
Guinea	0.015	163
Oman	0.012	164
Lithuania	0.011	165
New Caledonia	0.006	166
Norway	0.004	167
Netherlands Antilles	0.003	168
Mongolia	0.003	169
Liberia	0.002	170
United States Virgin Islands	0.002	171
Brunei	0.001	172
Sweden	0.001	173
United Arab Emirates	0.001	174
Kuwait	0.000	175
Qatar	0.000	176
Suriname	0.000	177
Bahrain	0.000	178
Namibia	0.000	179
Libya	0.000	180
American Samoa	0.000	181
Bahamas	0.000	181
British Virgin Islands	0.000	181
Cape Verde	0.000	181
Cayman Islands	0.000	181
Channel Islands [10]	0.000	181
Cook Islands	0.000	181
Cyprus	0.000	181
Denmark	0.000	181
Equatorial Guinea	0.000	181
Estonia	0.000	181
Faeroe Islands	0.000	181
Falkland Islands	0.000	181
Finland [11]	0.000	181
French Guiana	0.000	181
French Polynesia	0.000	181
Gambia	0.000	181

Table S6. *Cont.*

Country	Flood Exposure	Flood Rank
Gibraltar	0.000	181
Greenland	0.000	181
Grenada	0.000	181
Guinea-Bissau	0.000	181
Iceland	0.000	181
Isle of Man	0.000	181
Kiribati	0.000	181
Latvia	0.000	181
Maldives	0.000	181
Malta	0.000	181
Marshall Islands	0.000	181
Mauritius	0.000	181
Micronesia, Federated States of	0.000	181
Montserrat	0.000	181
Nauru	0.000	181
Niue	0.000	181
Northern Mariana Islands	0.000	181
Palau	0.000	181
Pitcairn Islands	0.000	181
Réunion	0.000	181
Saint Helena	0.000	181
Saint Kitts and Nevis	0.000	181
Saint Pierre and Miquelon	0.000	181
Saint Vincent and the Grenadines	0.000	181
Sao Tome and Principe	0.000	181
Seychelles	0.000	181
Tokelau	0.000	181
Tonga	0.000	181
Turks and Caicos Islands	0.000	181
Tuvalu	0.000	181
Wallis and Futuna Islands	0.000	181

Notes: [1] Special Administrative Region (SAR) of China; [2] Republic of Korea; [3] includes Christmas Island and Cocos islands; [4] Democratic People's Republic of Korea; [5] includes Saint Barthelemy and Saint Martin; [6] includes Southern Sudan; [7] includes Western Sahara; [8] Holy See; [9] includes Mayotte; [10] includes Guernsey and Jersey; [11] includes Aland Islands.

Table S7. Urban population exposure by country.

Country	% Rural	% Urban	Urban Per Capita Cyclone Exposure	Urban Per Capita Drought Exposure	Urban Per Capita Flood Exposure	Urban Per Capita Multi-Hazard Exposure
Afghanistan	76.5	23.5	0.000	0.746	0.725	1.472
Albania	46.6	53.4	0.000	0.001	0.159	0.160
Algeria	27.0	73.0	0.000	0.382	0.434	0.816
American Samoa	6.8	93.2	0.080	0.098	0.000	0.178
Andorra	12.7	87.3	0.000	0.094	0.100	0.194
Angola	40.8	59.2	0.000	0.706	0.147	0.853
Anguilla	N/A	100.0	0.839	0.500	0.037	1.376
Antigua and Barbuda	70.2	29.8	0.893	0.397	0.099	1.389
Argentina	7.5	92.5	0.000	0.302	0.657	0.959
Armenia	35.9	64.1	0.000	0.052	0.018	0.070
Aruba	53.2	46.9	0.017	0.067	0.102	0.186
Australia [1]	10.8	89.2	0.032	0.279	0.435	0.746
Austria	32.3	67.7	0.000	0.000	0.423	0.423
Azerbaijan	46.4	53.6	0.000	0.486	0.072	0.558
Bahamas	15.7	84.3	0.546	0.003	0.000	0.549
Bahrain	11.3	88.7	0.000	0.000	0.000	0.000
Bangladesh	71.6	28.4	0.165	0.556	0.999	1.720
Barbados	55.6	44.4	0.101	0.505	0.101	0.707
Belarus	25.0	75.0	0.000	0.000	0.157	0.157
Belgium	2.5	97.5	0.000	0.021	0.534	0.556
Belize	55.3	44.7	0.807	0.000	0.396	1.203
Benin	55.1	44.9	0.000	0.277	0.421	0.698
Bermuda	N/A	100.0	0.784	0.000	0.100	0.884
Bhutan	64.4	35.6	0.000	0.649	0.525	1.174
Bolivia	33.2	66.8	0.000	0.235	0.649	0.884
Bosnia and Herzegovina	51.7	48.3	0.000	0.020	0.272	0.292
Botswana	38.4	61.7	0.000	0.528	0.070	0.598
Brazil	15.4	84.6	0.000	0.237	0.610	0.846
British Virgin Islands	59.4	40.6	0.934	0.505	0.000	1.439
Brunei	24.0	76.0	0.000	0.103	0.000	0.103
Bulgaria	26.9	73.1	0.000	0.056	0.058	0.114
Burkina Faso	73.5	26.5	0.000	0.363	0.324	0.687
Burundi	89.1	10.9	0.000	0.156	0.498	0.654
Cambodia	80.0	20.0	0.001	0.251	0.844	1.097
Cameroon	47.9	52.1	0.000	0.143	0.241	0.384
Canada	19.4	80.7	0.095	0.125	0.083	0.303
Cape Verde	37.4	62.6	0.006	0.370	0.000	0.376

Table S7. *Cont.*

Country	% Rural	% Urban	Urban Per Capita Cyclone Exposure	Urban Per Capita Drought Exposure	Urban Per Capita Flood Exposure	Urban Per Capita Multi-Hazard Exposure
Cayman Islands	N/A	100.0	0.526	0.005	0.000	0.530
Central African Republic	60.9	39.1	0.000	0.144	0.442	0.586
Chad	78.2	21.8	0.000	0.378	0.242	0.620
Channel Islands [2]	68.8	31.2	0.060	0.119	0.000	0.179
Chile	10.8	89.2	0.000	0.751	0.670	1.421
China	49.4	50.6	0.249	0.322	0.577	1.148
Colombia	24.7	75.3	0.001	0.479	0.855	1.335
Comoros [3]	67.2	32.8	0.355	0.098	0.025	0.478
Congo, Republic of the	36.3	63.7	0.000	0.166	0.229	0.394
Cook Islands	26.5	73.5	0.214	0.178	0.000	0.392
Costa Rica	35.3	64.7	0.098	0.198	0.506	0.802
Côte d'Ivoire	48.7	51.3	0.000	0.064	0.045	0.109
Croatia	42.2	57.8	0.000	0.017	0.103	0.120
Cuba	24.8	75.2	0.498	0.262	0.467	1.226
Cyprus	29.5	70.5	0.000	0.473	0.000	0.473
Czech Republic	26.6	73.4	0.000	0.000	0.539	0.539
Democratic Republic of the Congo	65.7	34.3	0.000	0.160	0.275	0.435
Denmark	13.1	86.9	0.000	0.079	0.000	0.079
Djibouti	22.9	77.1	0.000	0.892	0.015	0.907
Dominica	32.9	67.1	0.798	0.399	0.096	1.293
Dominican Republic	30.3	69.7	0.536	0.044	0.796	1.376
Ecuador	32.5	67.5	0.000	0.676	0.854	1.530
Egypt	56.5	43.5	0.000	0.000	0.065	0.065
El Salvador	35.2	64.8	0.002	0.003	0.900	0.905
Equatorial Guinea	60.5	39.5	0.000	0.116	0.000	0.116
Eritrea	78.7	21.3	0.000	0.831	0.000	0.831
Estonia	30.5	69.5	0.000	0.001	0.000	0.001
Ethiopia	83.0	17.0	0.000	0.611	0.269	0.879
Faeroe Islands	58.9	41.1	0.000	0.000	0.000	0.000
Falkland Islands	25.9	74.1	0.000	0.000	0.000	0.000
Fiji	47.8	52.2	0.786	0.262	0.334	1.382
Finland [4]	16.3	83.7	0.000	0.011	0.000	0.011
France	14.2	85.8	0.007	0.038	0.550	0.595
French Guiana	23.6	76.4	0.000	0.000	0.000	0.000
French Polynesia	48.6	51.4	0.649	0.000	0.000	0.649
Gabon	13.8	86.2	0.000	0.091	0.030	0.121

Table S7. *Cont.*

Country	% Rural	% Urban	Urban Per Capita Cyclone Exposure	Urban Per Capita Drought Exposure	Urban Per Capita Flood Exposure	Urban Per Capita Multi-Hazard Exposure
Gambia	42.7	57.3	0.000	0.412	0.000	0.412
Georgia	47.2	52.8	0.000	0.010	0.378	0.388
Germany	26.1	73.9	0.000	0.053	0.602	0.655
Ghana	48.1	51.9	0.000	0.058	0.325	0.382
Gibraltar	N/A	100.0	0.000	1.000	0.000	1.000
Greece	38.6	61.4	0.000	0.561	0.289	0.849
Greenland	15.3	84.7	0.000	0.049	0.000	0.049
Grenada	60.9	39.1	0.334	0.000	0.000	0.334
Guadeloupe [5]	1.6	98.4	0.829	0.408	0.355	1.592
Guam	6.8	93.2	1.000	0.400	0.100	1.500
Guatemala	50.2	49.8	0.085	0.880	0.973	1.938
Guinea	64.6	35.4	0.000	0.515	0.009	0.524
Guinea-Bissau	56.1	43.9	0.000	0.226	0.000	0.226
Guyana	71.6	28.4	0.000	0.200	0.004	0.205
Haiti	46.6	53.4	0.432	0.067	0.912	1.410
Honduras	47.8	52.2	0.113	0.065	0.905	1.083
Hong Kong [6]	N/A	100.0	0.993	0.095	0.997	2.085
Hungary	30.5	69.5	0.000	0.026	0.300	0.326
Iceland	6.3	93.7	0.000	0.000	0.000	0.000
India	68.7	31.3	0.058	0.652	0.671	1.381
Indonesia	49.3	50.7	0.006	0.424	0.761	1.192
Iran	30.9	69.1	0.000	0.659	0.560	1.219
Iraq	33.5	66.5	0.000	0.712	0.051	0.763
Ireland	37.8	62.2	0.079	0.004	0.235	0.318
Isle of Man	49.5	50.5	0.000	0.000	0.000	0.000
Israel	8.1	91.9	0.000	0.335	0.424	0.759
Italy	31.6	68.4	0.000	0.083	0.348	0.432
Jamaica	48.0	52.0	0.239	0.016	0.980	1.236
Japan	8.7	91.3	0.977	0.009	0.461	1.446
Jordan	17.3	82.7	0.000	0.875	0.281	1.156
Kazakhstan	46.4	53.6	0.000	0.465	0.023	0.488
Kenya	76.0	24.0	0.000	0.253	0.880	1.134
Kiribati	56.1	43.9	0.000	0.700	0.000	0.700
Kuwait	1.8	98.3	0.000	0.000	0.000	0.000
Kyrgyzstan	64.7	35.4	0.000	0.482	0.085	0.568
Laos	65.8	34.3	0.262	0.487	0.547	1.295
Latvia	32.3	67.7	0.000	0.000	0.000	0.000
Lebanon	12.8	87.3	0.000	0.900	0.607	1.507
Lesotho	72.4	27.6	0.000	0.686	0.168	0.854
Liberia	51.8	48.2	0.000	0.169	0.001	0.170
Libya	22.3	77.7	0.000	0.441	0.000	0.441

Table S7. *Cont.*

Country	% Rural	% Urban	Urban Per Capita Cyclone Exposure	Urban Per Capita Drought Exposure	Urban Per Capita Flood Exposure	Urban Per Capita Multi-Hazard Exposure
Liechtenstein	85.6	14.4	0.000	0.000	0.755	0.755
Lithuania	32.9	67.1	0.000	0.000	0.013	0.013
Luxembourg	14.6	85.4	0.000	0.002	0.768	0.769
Macao [6]	N/A	100.0	0.895	0.100	1.000	1.995
Macedonia	40.7	59.3	0.000	0.009	0.445	0.454
Madagascar	67.4	32.6	0.882	0.199	0.372	1.452
Malawi	84.3	15.7	0.116	0.727	0.472	1.315
Malaysia	27.2	72.8	0.000	0.029	0.685	0.714
Maldives	58.8	41.2	0.000	0.000	0.000	0.000
Mali	65.1	34.9	0.000	0.412	0.121	0.532
Malta	5.2	94.8	0.000	0.900	0.000	0.900
Marshall Islands	28.2	71.8	0.000	0.000	0.000	0.000
Martinique	11.0	89.0	0.138	0.000	0.015	0.152
Mauritania	58.5	41.5	0.000	0.185	0.014	0.200
Mauritius	58.2	41.8	1.004	0.000	0.000	1.004
Mexico	21.9	78.1	0.092	0.532	0.552	1.176
Micronesia, Federated States of	77.4	22.6	0.649	0.395	0.000	1.045
Moldova, Republic of	52.3	47.7	0.000	0.154	0.149	0.303
Monaco	N/A	100.0	0.000	0.100	0.400	0.500
Mongolia	31.5	68.5	0.000	0.328	0.002	0.330
Montenegro	36.7	63.3	0.000	0.000	0.248	0.248
Montserrat	85.8	14.2	1.274	0.637	0.000	1.911
Morocco [7]	42.6	57.5	0.000	0.700	0.168	0.868
Mozambique	68.8	31.2	0.287	0.588	0.537	1.412
Myanmar	67.4	32.6	0.031	0.851	0.081	0.963
Namibia	61.6	38.4	0.000	0.646	0.000	0.646
Nauru	N/A	100.0	0.000	0.900	0.000	0.900
Nepal	83.0	17.0	0.000	0.575	0.983	1.558
Netherlands	16.8	83.2	0.000	0.056	0.271	0.327
Netherlands Antilles	6.6	93.4	0.224	0.426	0.001	0.651
New Caledonia	38.3	61.7	0.987	0.400	0.004	1.392
New Zealand	13.8	86.2	0.022	0.017	0.474	0.513
Nicaragua	42.5	57.5	0.126	0.634	0.688	1.449
Niger	82.2	17.8	0.000	0.411	0.707	1.118
Nigeria	50.4	49.6	0.000	0.465	0.343	0.807
Niue	62.1	37.9	0.466	0.187	0.000	0.653

Table S7. *Cont.*

Country	% Rural	% Urban	Urban Per Capita Cyclone Exposure	Urban Per Capita Drought Exposure	Urban Per Capita Flood Exposure	Urban Per Capita Multi-Hazard Exposure
North Korea [8]	39.7	60.3	0.435	0.218	0.354	1.007
Northern Mariana Islands	8.5	91.5	0.998	0.224	0.000	1.222
Norway	20.6	79.4	0.000	0.062	0.004	0.066
Oman	26.6	73.4	0.000	0.011	0.011	0.022
Pakistan	26.9	73.1	0.014	0.787	0.566	1.366
Palau	63.8	36.2	0.098	0.392	0.000	0.490
Palestine	25.7	74.4	0.000	0.466	0.358	0.824
Panama	15.8	84.3	0.002	0.045	0.432	0.478
Papua New Guinea	24.7	75.3	0.004	0.160	0.024	0.188
Paraguay	87.5	12.5	0.000	0.082	0.874	0.956
Peru	38.1	61.9	0.000	0.165	0.655	0.820
Philippines	22.7	77.3	0.888	0.394	0.832	2.114
Pitcairn Islands	51.2	48.8	0.000	0.000	0.000	0.000
Poland	39.1	60.9	0.000	0.035	0.306	0.341
Portugal	38.9	61.1	0.006	0.399	0.336	0.742
Puerto Rico	1.1	98.9	0.843	0.000	0.557	1.400
Qatar	1.2	98.8	0.000	0.000	0.000	0.000
Réunion	5.7	94.3	0.995	0.000	0.000	0.995
Romania	47.2	52.8	0.000	0.047	0.648	0.695
Russia	26.2	73.8	0.012	0.098	0.109	0.219
Rwanda	80.9	19.1	0.000	0.006	0.809	0.815
Saint Helena	60.5	39.5	0.000	0.000	0.000	0.000
Saint Kitts and Nevis	68.0	32.0	0.796	0.895	0.000	1.691
Saint Lucia	82.5	17.5	0.091	0.000	0.091	0.181
Saint Pierre and Miquelon	9.3	90.7	0.712	0.000	0.000	0.712
Saint Vincent and the Grenadines	50.7	49.3	0.413	0.000	0.000	0.413
Samoa	80.1	19.9	0.124	0.099	0.099	0.322
San Marino	5.9	94.1	0.000	0.000	0.100	0.100
Sao Tome and Principe	37.3	62.7	0.000	0.000	0.000	0.000
Saudi Arabia	17.7	82.3	0.000	0.021	0.134	0.155
Senegal	57.5	42.5	0.000	0.412	0.090	0.502
Serbia	43.6	56.4	0.000	0.010	0.546	0.556
Seychelles	46.4	53.6	0.000	0.000	0.000	0.000
Sierra Leone	60.8	39.2	0.000	0.671	0.034	0.705
Singapore	N/A	100.0	0.000	0.000	0.900	0.900

Table S7. *Cont.*

Country	% Rural	% Urban	Urban Per Capita Cyclone Exposure	Urban Per Capita Drought Exposure	Urban Per Capita Flood Exposure	Urban Per Capita Multi-Hazard Exposure
Slovakia	45.3	54.7	0.000	0.000	0.484	0.484
Slovenia	50.1	49.9	0.000	0.000	0.055	0.055
Solomon Islands	79.5	20.5	0.039	0.059	0.052	0.150
Somalia	62.3	37.7	0.000	0.867	0.632	1.500
South Africa	38.0	62.0	0.000	0.514	0.352	0.867
South Korea [9]	16.8	83.2	0.786	0.166	0.874	1.825
Spain	22.6	77.4	0.001	0.404	0.214	0.619
Sri Lanka	84.9	15.1	0.011	0.051	0.803	0.866
Sudan [10]	70.3	29.7	0.000	0.326	0.495	0.821
Suriname	30.3	69.7	0.000	0.000	0.000	0.000
Swaziland	78.8	21.3	0.001	0.901	0.196	1.098
Sweden	14.8	85.2	0.000	0.115	0.001	0.116
Switzerland	26.3	73.7	0.000	0.000	0.749	0.749
Syria	43.9	56.1	0.000	0.867	0.261	1.129
Tajikistan	73.5	26.5	0.000	0.680	0.671	1.351
Tanzania, United Republic of	73.3	26.7	0.000	0.325	0.509	0.834
Thailand	65.9	34.1	0.013	0.483	0.765	1.262
Timor-Leste	71.7	28.3	0.021	0.512	0.135	0.669
Togo	62.0	38.0	0.000	0.154	0.150	0.304
Tokelau	100.0	N/A	0.000	0.000	0.000	N/A
Tonga	76.6	23.4	0.567	0.409	0.000	0.976
Trinidad and Tobago	86.3	13.7	0.000	0.000	0.627	0.627
Tunisia	33.7	66.3	0.000	0.308	0.267	0.575
Turkey	28.5	71.5	0.000	0.369	0.289	0.658
Turkmenistan	51.3	48.7	0.000	0.112	0.156	0.268
Turks and Caicos Islands	6.2	93.8	0.728	0.000	0.000	0.728
Tuvalu	49.4	50.6	0.096	0.096	0.000	0.193
Uganda	84.4	15.6	0.000	0.013	0.754	0.767
Ukraine	31.1	68.9	0.000	0.030	0.134	0.164
United Arab Emirates	15.6	84.4	0.000	0.689	0.000	0.689
United Kingdom	20.4	79.6	0.062	0.033	0.817	0.912
United States of America	17.6	82.4	0.237	0.228	0.520	0.986
United States Virgin Islands	4.6	95.5	0.850	0.498	0.001	1.349
Uruguay	7.5	92.6	0.000	0.107	0.497	0.604
Uzbekistan	63.8	36.2	0.000	0.495	0.310	0.805

Table S7. *Cont.*

Country	% Rural	% Urban	Urban Per Capita Cyclone Exposure	Urban Per Capita Drought Exposure	Urban Per Capita Flood Exposure	Urban Per Capita Multi-Hazard Exposure
Vanuatu	75.1	24.9	0.895	0.363	0.127	1.385
Vatican City State [11]	N/A	100.0	0.000	0.100	0.100	0.200
Venezuela	6.5	93.5	0.000	0.402	0.635	1.037
Vietnam	69.0	31.0	0.475	0.372	0.877	1.725
Wallis and Futuna Islands	100.0	N/A	0.000	0.000	0.000	N/A
Yemen	67.7	32.3	0.000	0.000	0.625	0.625
Zambia	60.9	39.2	0.000	0.655	0.007	0.662
Zimbabwe	61.4	38.6	0.000	0.878	0.132	1.010

Notes: [1] includes Christmas Island and Cocos islands; [2] includes Guernsey and Jersey; [3] includes Mayotte; [4] includes Aland Islands; [5] includes Saint Barthelemy and Saint Martin; [6] Special Administrative Region (SAR) of China; [7] includes Western Sahara; [8] Democratic People's Republic of Korea; [9] Republic of Korea; [10] includes Southern Sudan; [11] Holy See.

Table S8. Rural population exposure by country.

Country	% Rural	% Urban	Rural Per Capita Cyclone Exposure	Rural Per Capita Drought Exposure	Rural Per Capita Flood Exposure	Rural Per Capita Multi-Hazard Exposure
Afghanistan	76.5	23.5	0.000	0.569	0.518	1.087
Albania	46.6	53.4	0.000	0.026	0.192	0.219
Algeria	27.0	73.0	0.000	0.331	0.403	0.734
American Samoa	6.8	93.2	0.087	0.090	0.000	0.177
Andorra	12.7	87.3	0.000	0.083	0.101	0.184
Angola	40.8	59.2	0.000	0.610	0.051	0.661
Anguilla	N/A	N/A	0.000	0.000	0.000	N/A
Antigua and Barbuda	70.2	29.8	0.903	0.401	0.099	1.403
Argentina	7.5	92.5	0.000	0.275	0.451	0.727
Armenia	35.9	64.1	0.000	0.172	0.063	0.235
Aruba	53.2	46.9	0.022	0.112	0.098	0.232
Australia [1]	10.8	89.2	0.031	0.222	0.222	0.475
Austria	32.3	67.7	0.000	0.000	0.381	0.381
Azerbaijan	46.4	53.6	0.000	0.486	0.084	0.570
Bahamas	15.7	84.3	0.661	0.045	0.000	0.706
Bahrain	11.3	88.7	0.000	0.000	0.001	0.001
Bangladesh	71.6	28.4	0.149	0.653	0.999	1.802
Barbados	55.6	44.4	0.099	0.496	0.098	0.693
Belarus	25.0	75.0	0.000	0.000	0.222	0.222

Table S8. *Cont.*

Country	% Rural	% Urban	Rural Per Capita Cyclone Exposure	Rural Per Capita Drought Exposure	Rural Per Capita Flood Exposure	Rural Per Capita Multi-Hazard Exposure
Belgium	2.5	97.5	0.000	0.031	0.593	0.624
Belize	55.3	44.7	0.714	0.000	0.414	1.128
Benin	55.1	44.9	0.000	0.346	0.453	0.799
Bermuda	N/A	N/A	0.000	0.000	0.000	N/A
Bhutan	64.4	35.6	0.000	0.726	0.532	1.257
Bolivia	33.2	66.8	0.000	0.245	0.562	0.808
Bosnia and Herzegovina	51.7	48.3	0.000	0.027	0.267	0.294
Botswana	38.4	61.7	0.000	0.501	0.098	0.598
Brazil	15.4	84.6	0.000	0.385	0.315	0.700
British Virgin Islands	59.4	40.6	0.898	0.497	0.000	1.395
Brunei	24.0	76.0	0.000	0.104	0.006	0.110
Bulgaria	26.9	73.1	0.000	0.051	0.082	0.133
Burkina Faso	73.5	26.5	0.000	0.324	0.282	0.606
Burundi	89.1	10.9	0.000	0.227	0.572	0.799
Cambodia	80.0	20.0	0.006	0.396	0.879	1.281
Cameroon	47.9	52.1	0.000	0.145	0.141	0.286
Canada	19.4	80.7	0.114	0.120	0.061	0.296
Cape Verde	37.4	62.6	0.021	0.423	0.000	0.444
Cayman Islands	N/A	N/A	0.000	0.000	0.000	N/A
Central African Republic	60.9	39.1	0.000	0.189	0.226	0.415
Chad	78.2	21.8	0.000	0.436	0.233	0.669
Channel Islands [2]	68.8	31.2	0.032	0.063	0.000	0.095
Chile	10.8	89.2	0.000	0.435	0.355	0.790
China	49.4	50.6	0.154	0.364	0.527	1.045
Colombia	24.7	75.3	0.001	0.331	0.843	1.175
Comoros [3]	67.2	32.8	0.400	0.124	0.043	0.567
Congo, Republic of the	36.3	63.7	0.000	0.196	0.145	0.341
Cook Islands	26.5	73.5	0.235	0.181	0.000	0.416
Costa Rica	35.3	64.7	0.086	0.255	0.679	1.020
Côte d'Ivoire	48.7	51.3	0.000	0.176	0.022	0.198
Croatia	42.2	57.8	0.000	0.019	0.125	0.144
Cuba	24.8	75.2	0.468	0.272	0.647	1.387
Cyprus	29.5	70.5	0.000	0.514	0.000	0.514
Czech Republic	26.6	73.4	0.000	0.000	0.496	0.496
Democratic Republic of the Congo	65.7	34.3	0.000	0.127	0.180	0.307

Table S8. *Cont.*

Country	% Rural	% Urban	Rural Per Capita Cyclone Exposure	Rural Per Capita Drought Exposure	Rural Per Capita Flood Exposure	Rural Per Capita Multi-Hazard Exposure
Denmark	13.1	86.9	0.000	0.119	0.000	0.119
Djibouti	22.9	77.1	0.000	0.824	0.117	0.941
Dominica	32.9	67.1	0.804	0.402	0.097	1.303
Dominican Republic	30.3	69.7	0.559	0.071	0.786	1.416
Ecuador	32.5	67.5	0.000	0.599	0.800	1.399
Egypt	56.5	43.5	0.000	0.000	0.015	0.015
El Salvador	35.2	64.8	0.009	0.017	0.897	0.923
Equatorial Guinea	60.5	39.5	0.000	0.036	0.000	0.036
Eritrea	78.7	21.3	0.000	0.790	0.049	0.839
Estonia	30.5	69.5	0.000	0.002	0.000	0.002
Ethiopia	83.0	17.0	0.000	0.544	0.222	0.765
Faeroe Islands	58.9	41.1	0.000	0.000	0.000	0.000
Falkland Islands	25.9	74.1	0.000	0.001	0.000	0.001
Fiji	47.8	52.2	0.738	0.234	0.285	1.257
Finland [4]	16.3	83.7	0.000	0.018	0.000	0.018
France	14.2	85.8	0.012	0.047	0.423	0.482
French Guiana	23.6	76.4	0.000	0.000	0.000	0.000
French Polynesia	48.6	51.4	0.337	0.066	0.000	0.403
Gabon	13.8	86.2	0.000	0.076	0.049	0.125
Gambia	42.7	57.3	0.000	0.436	0.000	0.436
Georgia	47.2	52.8	0.000	0.036	0.331	0.367
Germany	26.1	73.9	0.000	0.045	0.582	0.627
Ghana	48.1	51.9	0.000	0.173	0.187	0.360
Gibraltar	N/A	N/A	0.000	0.000	0.000	N/A
Greece	38.6	61.4	0.000	0.489	0.138	0.628
Greenland	15.3	84.7	0.000	0.014	0.000	0.014
Grenada	60.9	39.1	0.148	0.000	0.000	0.148
Guadeloupe [5]	1.6	98.4	0.814	0.400	0.325	1.539
Guam	6.8	93.2	0.999	0.400	0.100	1.498
Guatemala	50.2	49.8	0.068	0.804	0.936	1.808
Guinea	64.6	35.4	0.000	0.273	0.018	0.292
Guinea-Bissau	56.1	43.9	0.000	0.299	0.000	0.299
Guyana	71.6	28.4	0.000	0.190	0.022	0.212
Haiti	46.6	53.4	0.371	0.047	0.830	1.248
Honduras	47.8	52.2	0.115	0.070	0.884	1.069
Hong Kong [6]	N/A	N/A	0.000	0.000	0.000	N/A
Hungary	30.5	69.5	0.000	0.027	0.380	0.407
Iceland	6.3	93.7	0.000	0.000	0.000	0.000
India	68.7	31.3	0.044	0.653	0.580	1.277

Table S8. *Cont.*

Country	% Rural	% Urban	Rural Per Capita Cyclone Exposure	Rural Per Capita Drought Exposure	Rural Per Capita Flood Exposure	Rural Per Capita Multi-Hazard Exposure
Indonesia	49.3	50.7	0.006	0.426	0.639	1.071
Iran	30.9	69.1	0.000	0.671	0.503	1.175
Iraq	33.5	66.5	0.000	0.735	0.054	0.789
Ireland	37.8	62.2	0.066	0.005	0.099	0.170
Isle of Man	49.5	50.5	0.000	0.000	0.000	0.000
Israel	8.1	91.9	0.000	0.467	0.508	0.975
Italy	31.6	68.4	0.000	0.095	0.324	0.419
Jamaica	48.0	52.0	0.166	0.040	0.951	1.156
Japan	8.7	91.3	0.959	0.011	0.291	1.261
Jordan	17.3	82.7	0.000	0.817	0.331	1.148
Kazakhstan	46.4	53.6	0.000	0.392	0.052	0.444
Kenya	76.0	24.0	0.000	0.319	0.805	1.125
Kiribati	56.1	43.9	0.000	0.715	0.000	0.715
Kuwait	1.8	98.3	0.000	0.046	0.024	0.070
Kyrgyzstan	64.7	35.4	0.000	0.436	0.087	0.523
Laos	65.8	34.3	0.270	0.551	0.523	1.345
Latvia	32.3	67.7	0.000	0.001	0.000	0.001
Lebanon	12.8	87.3	0.000	0.900	0.552	1.451
Lesotho	72.4	27.6	0.000	0.807	0.299	1.105
Liberia	51.8	48.2	0.000	0.094	0.003	0.097
Libya	22.3	77.7	0.000	0.417	0.000	0.417
Liechtenstein	85.6	14.4	0.000	0.000	0.924	0.924
Lithuania	32.9	67.1	0.000	0.000	0.008	0.008
Luxembourg	14.6	85.4	0.000	0.008	0.768	0.776
Macao [6]	N/A	N/A	0.000	0.000	0.000	N/A
Macedonia	40.7	59.3	0.000	0.034	0.433	0.466
Madagascar	67.4	32.6	0.838	0.257	0.238	1.333
Malawi	84.3	15.7	0.075	0.697	0.432	1.204
Malaysia	27.2	72.8	0.000	0.047	0.638	0.685
Maldives	58.8	41.2	0.000	0.000	0.000	0.000
Mali	65.1	34.9	0.000	0.358	0.114	0.472
Malta	5.2	94.8	0.000	0.897	0.000	0.897
Marshall Islands	28.2	71.8	0.000	0.000	0.000	0.000
Martinique	11.0	89.0	0.334	0.000	0.057	0.391
Mauritania	58.5	41.5	0.000	0.163	0.029	0.192
Mauritius	58.2	41.8	0.985	0.042	0.000	1.027
Mexico	21.9	78.1	0.137	0.511	0.497	1.145
Micronesia, Federated States of	77.4	22.6	0.632	0.401	0.000	1.033

Table S8. *Cont.*

Country	% Rural	% Urban	Rural Per Capita Cyclone Exposure	Rural Per Capita Drought Exposure	Rural Per Capita Flood Exposure	Rural Per Capita Multi-Hazard Exposure
Moldova, Republic of	52.3	47.7	0.000	0.122	0.174	0.297
Monaco	N/A	N/A	0.000	0.000	0.000	N/A
Mongolia	31.5	68.5	0.000	0.078	0.004	0.082
Montenegro	36.7	63.3	0.000	0.000	0.226	0.226
Montserrat	85.8	14.2	0.767	0.361	0.000	1.128
Morocco [7]	42.6	57.5	0.000	0.661	0.128	0.789
Mozambique	68.8	31.2	0.386	0.627	0.364	1.378
Myanmar	67.4	32.6	0.032	0.847	0.092	0.970
Namibia	61.6	38.4	0.000	0.558	0.000	0.558
Nauru	N/A	N/A	0.000	0.000	0.000	N/A
Nepal	83.0	17.0	0.000	0.503	0.948	1.452
Netherlands	16.8	83.2	0.000	0.069	0.293	0.362
Netherlands Antilles	6.6	93.4	0.144	0.415	0.032	0.591
New Caledonia	38.3	61.7	0.957	0.419	0.008	1.384
New Zealand	13.8	86.2	0.030	0.013	0.270	0.314
Nicaragua	42.5	57.5	0.195	0.306	0.675	1.176
Niger	82.2	17.8	0.000	0.388	0.688	1.076
Nigeria	50.4	49.6	0.000	0.437	0.305	0.742
Niue	62.1	37.9	0.545	0.208	0.000	0.753
North Korea [8]	39.7	60.3	0.457	0.227	0.426	1.109
Northern Mariana Islands	8.5	91.5	1.022	0.284	0.000	1.306
Norway	20.6	79.4	0.000	0.046	0.005	0.051
Oman	26.6	73.4	0.000	0.025	0.014	0.039
Pakistan	26.9	73.1	0.007	0.750	0.472	1.229
Palau	63.8	36.2	0.136	0.400	0.000	0.536
Palestine	25.7	74.4	0.000	0.754	0.442	1.196
Panama	15.8	84.3	0.002	0.082	0.371	0.455
Papua New Guinea	24.7	75.3	0.007	0.204	0.017	0.227
Paraguay	87.5	12.5	0.000	0.097	0.798	0.894
Peru	38.1	61.9	0.000	0.190	0.643	0.834
Philippines	22.7	77.3	0.807	0.390	0.711	1.909
Pitcairn Islands	51.2	48.8	0.000	0.000	0.000	0.000
Poland	39.1	60.9	0.000	0.032	0.326	0.358
Portugal	38.9	61.1	0.008	0.353	0.388	0.749
Puerto Rico	1.1	98.9	0.818	0.000	0.578	1.395
Qatar	1.2	98.8	0.000	0.000	0.002	0.002
Réunion	5.7	94.3	0.993	0.000	0.000	0.993

Table S8. *Cont.*

Country	% Rural	% Urban	Rural Per Capita Cyclone Exposure	Rural Per Capita Drought Exposure	Rural Per Capita Flood Exposure	Rural Per Capita Multi-Hazard Exposure
Romania	47.2	52.8	0.000	0.040	0.674	0.714
Russia	26.2	73.8	0.006	0.132	0.093	0.231
Rwanda	80.9	19.1	0.000	0.013	0.820	0.833
Saint Helena	60.5	39.5	0.000	0.000	0.000	0.000
Saint Kitts and Nevis	68.0	32.0	0.802	0.902	0.000	1.704
Saint Lucia	82.5	17.5	0.166	0.000	0.042	0.207
Saint Pierre and Miquelon	9.3	90.7	0.573	0.000	0.000	0.573
Saint Vincent and the Grenadines	50.7	49.3	0.222	0.000	0.000	0.222
Samoa	80.1	19.9	0.237	0.092	0.059	0.388
San Marino	5.9	94.1	0.000	0.000	0.102	0.102
Sao Tome and Principe	37.3	62.7	0.000	0.000	0.000	0.000
Saudi Arabia	17.7	82.3	0.000	0.056	0.055	0.111
Senegal	57.5	42.5	0.000	0.431	0.068	0.499
Serbia	43.6	56.4	0.000	0.021	0.499	0.520
Seychelles	46.4	53.6	0.000	0.000	0.000	0.000
Sierra Leone	60.8	39.2	0.000	0.536	0.059	0.595
Singapore	N/A	N/A	0.000	0.000	0.000	N/A
Slovakia	45.3	54.7	0.000	0.000	0.505	0.505
Slovenia	50.1	49.9	0.000	0.000	0.078	0.078
Solomon Islands	79.5	20.5	0.217	0.054	0.060	0.330
Somalia	62.3	37.7	0.000	0.719	0.415	1.135
South Africa	38.0	62.0	0.001	0.522	0.478	1.001
South Korea [9]	16.8	83.2	0.748	0.149	0.819	1.716
Spain	22.6	77.4	0.002	0.485	0.154	0.641
Sri Lanka	84.9	15.1	0.058	0.108	0.894	1.060
Sudan [10]	70.3	29.7	0.000	0.523	0.229	0.751
Suriname	30.3	69.7	0.000	0.001	0.001	0.002
Swaziland	78.8	21.3	0.009	0.900	0.358	1.266
Sweden	14.8	85.2	0.000	0.103	0.001	0.104
Switzerland	26.3	73.7	0.000	0.000	0.757	0.757
Syria	43.9	56.1	0.000	0.801	0.219	1.020
Tajikistan	73.5	26.5	0.000	0.609	0.683	1.292
Tanzania, United Republic of	73.3	26.7	0.000	0.407	0.407	0.814
Thailand	65.9	34.1	0.034	0.598	0.841	1.473
Timor-Leste	71.7	28.3	0.054	0.524	0.209	0.787
Togo	62.0	38.0	0.000	0.287	0.296	0.583

Table S8. *Cont.*

Country	% Rural	% Urban	Rural Per Capita Cyclone Exposure	Rural Per Capita Drought Exposure	Rural Per Capita Flood Exposure	Rural Per Capita Multi-Hazard Exposure
Tokelau	100.0	N/A	0.000	0.000	0.000	0.000
Tonga	76.6	23.4	0.514	0.316	0.000	0.830
Trinidad and Tobago	86.3	13.7	0.010	0.000	0.657	0.667
Tunisia	33.7	66.3	0.000	0.253	0.383	0.636
Turkey	28.5	71.5	0.000	0.434	0.169	0.603
Turkmenistan	51.3	48.7	0.000	0.169	0.102	0.272
Turks and Caicos Islands	6.2	93.8	0.780	0.000	0.000	0.780
Tuvalu	49.4	50.6	0.007	0.661	0.000	0.668
Uganda	84.4	15.6	0.000	0.057	0.666	0.723
Ukraine	31.1	68.9	0.000	0.028	0.185	0.213
United Arab Emirates	15.6	84.4	0.000	0.453	0.002	0.456
United Kingdom	20.4	79.6	0.064	0.024	0.717	0.805
United States of America	17.6	82.4	0.233	0.117	0.514	0.864
United States Virgin Islands	4.6	95.5	0.861	0.493	0.010	1.363
Uruguay	7.5	92.6	0.000	0.113	0.625	0.738
Uzbekistan	63.8	36.2	0.000	0.406	0.299	0.705
Vanuatu	75.1	24.9	0.778	0.248	0.104	1.130
Vatican City State [11]	N/A	N/A	0.000	0.000	0.000	N/A
Venezuela	6.5	93.5	0.002	0.318	0.516	0.837
Vietnam	69.0	31.0	0.479	0.382	0.878	1.740
Wallis and Futuna Islands	100.0	N/A	0.647	0.200	0.000	0.847
Yemen	67.7	32.3	0.000	0.001	0.606	0.606
Zambia	60.9	39.2	0.000	0.592	0.027	0.619
Zimbabwe	61.4	38.6	0.000	0.773	0.194	0.967

Notes: [1] includes Christmas Island and Cocos islands; [2] includes Guernsey and Jersey; [3] includes Mayotte; [4] includes Aland Islands; [5] includes Saint Barthelemy and Saint Martin; [6] Special Administrative Region (SAR) of China; [7] includes Western Sahara; [8] Democratic People's Republic of Korea; [9] Republic of Korea; [10] includes Southern Sudan; [11] Holy See.

Table S9. Urban and rural population cyclone exposure.

ISO3	Country Name	Urban Per Capita Cyclone Exposure	Rural Per Capita Cyclone Exposure
AFG	Afghanistan	0.000	0.000
ALB	Albania	0.000	0.000
DZA	Algeria	0.000	0.000
ASM	American Samoa	0.080	0.087
AND	Andorra	0.000	0.000
AGO	Angola	0.000	0.000
AIA	Anguilla	0.839	0.000
ATG	Antigua and Barbuda	0.893	0.903
ARG	Argentina	0.000	0.000
ARM	Armenia	0.000	0.000
ABW	Aruba	0.017	0.022
AUS	Australia [1]	0.032	0.031
AUT	Austria	0.000	0.000
AZE	Azerbaijan	0.000	0.000
BHS	Bahamas	0.546	0.661
BHR	Bahrain	0.000	0.000
BGD	Bangladesh	0.165	0.149
BRB	Barbados	0.101	0.099
BLR	Belarus	0.000	0.000
BEL	Belgium	0.000	0.000
BLZ	Belize	0.807	0.714
BEN	Benin	0.000	0.000
BMU	Bermuda	0.784	0.000
BTN	Bhutan	0.000	0.000
BOL	Bolivia	0.000	0.000
BIH	Bosnia and Herzegovina	0.000	0.000
BWA	Botswana	0.000	0.000
BRA	Brazil	0.000	0.000
VGB	British Virgin Islands	0.934	0.898
BRN	Brunei	0.000	0.000
BGR	Bulgaria	0.000	0.000
BFA	Burkina Faso	0.000	0.000
BDI	Burundi	0.000	0.000
KHM	Cambodia	0.001	0.006
CMR	Cameroon	0.000	0.000
CAN	Canada	0.095	0.114
CPV	Cape Verde	0.006	0.021
CYM	Cayman Islands	0.526	0.000
CAF	Central African Republic	0.000	0.000
TCD	Chad	0.000	0.000
CHA	Channel Islands [2]	0.060	0.032
CHL	Chile	0.000	0.000
CHN	China	0.249	0.154

Table S9. *Cont.*

ISO3	Country Name	Urban Per Capita Cyclone Exposure	Rural Per Capita Cyclone Exposure
COL	Colombia	0.001	0.001
COM	Comoros [3]	0.355	0.400
COG	Congo, Republic of the	0.000	0.000
COK	Cook Islands	0.214	0.235
CRI	Costa Rica	0.098	0.086
CIV	Côte d'Ivoire	0.000	0.000
HRV	Croatia	0.000	0.000
CUB	Cuba	0.498	0.468
CYP	Cyprus	0.000	0.000
CZE	Czech Republic	0.000	0.000
COD	Democratic Republic of the Congo	0.000	0.000
DNK	Denmark	0.000	0.000
DJI	Djibouti	0.000	0.000
DMA	Dominica	0.798	0.804
DOM	Dominican Republic	0.536	0.559
ECU	Ecuador	0.000	0.000
EGY	Egypt	0.000	0.000
SLV	El Salvador	0.002	0.009
GNQ	Equatorial Guinea	0.000	0.000
ERI	Eritrea	0.000	0.000
EST	Estonia	0.000	0.000
ETH	Ethiopia	0.000	0.000
FRO	Faeroe Islands	0.000	0.000
FLK	Falkland Islands	0.000	0.000
FJI	Fiji	0.786	0.738
FIN	Finland [4]	0.000	0.000
FRA	France	0.007	0.012
GUF	French Guiana	0.000	0.000
PYF	French Polynesia	0.649	0.337
GAB	Gabon	0.000	0.000
GMB	Gambia	0.000	0.000
GEO	Georgia	0.000	0.000
DEU	Germany	0.000	0.000
GHA	Ghana	0.000	0.000
GIB	Gibraltar	0.000	0.000
GRC	Greece	0.000	0.000
GRL	Greenland	0.000	0.000
GRD	Grenada	0.334	0.148
GLP	Guadeloupe [5]	0.829	0.814
GUM	Guam	1.000	0.999
GTM	Guatemala	0.085	0.068
GIN	Guinea	0.000	0.000
GNB	Guinea-Bissau	0.000	0.000

Table S9. *Cont.*

ISO3	Country Name	Urban Per Capita Cyclone Exposure	Rural Per Capita Cyclone Exposure
GUY	Guyana	0.000	0.000
HTI	Haiti	0.432	0.371
HND	Honduras	0.113	0.115
HKG	Hong Kong [6]	0.993	0.000
HUN	Hungary	0.000	0.000
ISL	Iceland	0.000	0.000
IND	India	0.058	0.044
IDN	Indonesia	0.006	0.006
IRN	Iran	0.000	0.000
IRQ	Iraq	0.000	0.000
IRL	Ireland	0.079	0.066
IMN	Isle of Man	0.000	0.000
ISR	Israel	0.000	0.000
ITA	Italy	0.000	0.000
JAM	Jamaica	0.239	0.166
JPN	Japan	0.977	0.959
JOR	Jordan	0.000	0.000
KAZ	Kazakhstan	0.000	0.000
KEN	Kenya	0.000	0.000
KIR	Kiribati	0.000	0.000
KWT	Kuwait	0.000	0.000
KGZ	Kyrgyzstan	0.000	0.000
LAO	Laos	0.262	0.270
LVA	Latvia	0.000	0.000
LBN	Lebanon	0.000	0.000
LSO	Lesotho	0.000	0.000
LBR	Liberia	0.000	0.000
LBY	Libya	0.000	0.000
LIE	Liechtenstein	0.000	0.000
LTU	Lithuania	0.000	0.000
LUX	Luxembourg	0.000	0.000
MAC	Macao [6]	0.895	0.000
MKD	Macedonia	0.000	0.000
MDG	Madagascar	0.882	0.838
MWI	Malawi	0.116	0.075
MYS	Malaysia	0.000	0.000
MDV	Maldives	0.000	0.000
MLI	Mali	0.000	0.000
MLT	Malta	0.000	0.000
MHL	Marshall Islands	0.000	0.000
MTQ	Martinique	0.138	0.334
MRT	Mauritania	0.000	0.000
MUS	Mauritius	1.004	0.985

Table S9. *Cont.*

ISO3	Country Name	Urban Per Capita Cyclone Exposure	Rural Per Capita Cyclone Exposure
MEX	Mexico	0.092	0.137
FSM	Micronesia, Federated States of	0.649	0.632
MDA	Moldova, Republic of	0.000	0.000
MCO	Monaco	0.000	0.000
MNG	Mongolia	0.000	0.000
MNE	Montenegro	0.000	0.000
MSR	Montserrat	1.274	0.767
MAR	Morocco [7]	0.000	0.000
MOZ	Mozambique	0.287	0.386
MMR	Myanmar	0.031	0.032
NAM	Namibia	0.000	0.000
NRU	Nauru	0.000	0.000
NPL	Nepal	0.000	0.000
NLD	Netherlands	0.000	0.000
ANT	Netherlands Antilles	0.224	0.144
NCL	New Caledonia	0.987	0.957
NZL	New Zealand	0.022	0.030
NIC	Nicaragua	0.126	0.195
NER	Niger	0.000	0.000
NGA	Nigeria	0.000	0.000
NIU	Niue	0.466	0.545
PRK	North Korea [8]	0.435	0.457
MNP	Northern Mariana Islands	0.998	1.022
NOR	Norway	0.000	0.000
OMN	Oman	0.000	0.000
PAK	Pakistan	0.014	0.007
PLW	Palau	0.098	0.136
PSE	Palestine	0.000	0.000
PAN	Panama	0.002	0.002
PNG	Papua New Guinea	0.004	0.007
PRY	Paraguay	0.000	0.000
PER	Peru	0.000	0.000
PHL	Philippines	0.888	0.807
PCN	Pitcairn Islands	0.000	0.000
POL	Poland	0.000	0.000
PRT	Portugal	0.006	0.008
PRI	Puerto Rico	0.843	0.818
QAT	Qatar	0.000	0.000
REU	Réunion	0.995	0.993
ROU	Romania	0.000	0.000
RUS	Russia	0.012	0.006
RWA	Rwanda	0.000	0.000
SHN	Saint Helena	0.000	0.000

Table S9. *Cont.*

ISO3	Country Name	Urban Per Capita Cyclone Exposure	Rural Per Capita Cyclone Exposure
KNA	Saint Kitts and Nevis	0.796	0.802
LCA	Saint Lucia	0.091	0.166
SPM	Saint Pierre and Miquelon	0.712	0.573
VCT	Saint Vincent and the Grenadines	0.413	0.222
WSM	Samoa	0.124	0.237
SMR	San Marino	0.000	0.000
STP	Sao Tome and Principe	0.000	0.000
SAU	Saudi Arabia	0.000	0.000
SEN	Senegal	0.000	0.000
SRB	Serbia	0.000	0.000
SYC	Seychelles	0.000	0.000
SLE	Sierra Leone	0.000	0.000
SGP	Singapore	0.000	0.000
SVK	Slovakia	0.000	0.000
SVN	Slovenia	0.000	0.000
SLB	Solomon Islands	0.039	0.217
SOM	Somalia	0.000	0.000
ZAF	South Africa	0.000	0.001
KOR	South Korea [9]	0.786	0.748
ESP	Spain	0.001	0.002
LKA	Sri Lanka	0.011	0.058
SDN	Sudan [10]	0.000	0.000
SUR	Suriname	0.000	0.000
SWZ	Swaziland	0.001	0.009
SWE	Sweden	0.000	0.000
CHE	Switzerland	0.000	0.000
SYR	Syria	0.000	0.000
TJK	Tajikistan	0.000	0.000
TZA	Tanzania, United Republic of	0.000	0.000
THA	Thailand	0.013	0.034
TLS	Timor-Leste	0.021	0.054
TGO	Togo	0.000	0.000
TKL	Tokelau	0.000	0.000
TON	Tonga	0.567	0.514
TTO	Trinidad and Tobago	0.000	0.010
TUN	Tunisia	0.000	0.000
TUR	Turkey	0.000	0.000
TKM	Turkmenistan	0.000	0.000
TCA	Turks and Caicos Islands	0.728	0.780
TUV	Tuvalu	0.096	0.007
UGA	Uganda	0.000	0.000
UKR	Ukraine	0.000	0.000
ARE	United Arab Emirates	0.000	0.000

Table S9. *Cont.*

ISO3	Country Name	Urban Per Capita Cyclone Exposure	Rural Per Capita Cyclone Exposure
GBR	United Kingdom	0.062	0.064
USA	United States of America	0.237	0.233
VIR	United States Virgin Islands	0.850	0.861
URY	Uruguay	0.000	0.000
UZB	Uzbekistan	0.000	0.000
VUT	Vanuatu	0.895	0.778
VAT	Vatican City State [11]	0.000	0.000
VEN	Venezuela	0.000	0.002
VNM	Vietnam	0.475	0.479
WLF	Wallis and Futuna Islands	0.000	0.647
YEM	Yemen	0.000	0.000
ZMB	Zambia	0.000	0.000
ZWE	Zimbabwe	0.000	0.000

Notes: [1] includes Christmas Island and Cocos islands; [2] includes Guernsey and Jersey; [3] includes Mayotte; [4] includes Aland Islands; [5] includes Saint Barthelemy and Saint Martin; [6] Special Administrative Region (SAR) of China; [7] includes Western Sahara; [8] Democratic People's Republic of Korea; [9] Republic of Korea; [10] includes Southern Sudan; [11] Holy See.

Table S10. Urban and rural population drought exposure.

ISO3	Country Name	Urban Per Capita Drought Exposure	Rural Per Capita Drought Exposure
AFG	Afghanistan	0.746	0.569
ALB	Albania	0.001	0.026
DZA	Algeria	0.382	0.331
ASM	American Samoa	0.098	0.090
AND	Andorra	0.094	0.083
AGO	Angola	0.706	0.610
AIA	Anguilla	0.500	0.000
ATG	Antigua and Barbuda	0.397	0.401
ARG	Argentina	0.302	0.275
ARM	Armenia	0.052	0.172
ABW	Aruba	0.067	0.112
AUS	Australia [1]	0.279	0.222
AUT	Austria	0.000	0.000
AZE	Azerbaijan	0.486	0.486
BHS	Bahamas	0.003	0.045
BHR	Bahrain	0.000	0.000
BGD	Bangladesh	0.556	0.653
BRB	Barbados	0.505	0.496
BLR	Belarus	0.000	0.000
BEL	Belgium	0.021	0.031
BLZ	Belize	0.000	0.000

Table S10. *Cont.*

ISO3	Country Name	Urban Per Capita Drought Exposure	Rural Per Capita Drought Exposure
BEN	Benin	0.277	0.346
BMU	Bermuda	0.000	0.000
BTN	Bhutan	0.649	0.726
BOL	Bolivia	0.235	0.245
BIH	Bosnia and Herzegovina	0.020	0.027
BWA	Botswana	0.528	0.501
BRA	Brazil	0.237	0.385
VGB	British Virgin Islands	0.505	0.497
BRN	Brunei	0.103	0.104
BGR	Bulgaria	0.056	0.051
BFA	Burkina Faso	0.363	0.324
BDI	Burundi	0.156	0.227
KHM	Cambodia	0.251	0.396
CMR	Cameroon	0.143	0.145
CAN	Canada	0.125	0.120
CPV	Cape Verde	0.370	0.423
CYM	Cayman Islands	0.005	0.000
CAF	Central African Republic	0.144	0.189
TCD	Chad	0.378	0.436
CHA	Channel Islands [2]	0.119	0.063
CHL	Chile	0.751	0.435
CHN	China	0.322	0.364
COL	Colombia	0.479	0.331
COM	Comoros [3]	0.098	0.124
COG	Congo, Republic of the	0.166	0.196
COK	Cook Islands	0.178	0.181
CRI	Costa Rica	0.198	0.255
CIV	Côte d'Ivoire	0.064	0.176
HRV	Croatia	0.017	0.019
CUB	Cuba	0.262	0.272
CYP	Cyprus	0.473	0.514
CZE	Czech Republic	0.000	0.000
COD	Democratic Republic of the Congo	0.160	0.127
DNK	Denmark	0.079	0.119
DJI	Djibouti	0.892	0.824
DMA	Dominica	0.399	0.402
DOM	Dominican Republic	0.044	0.071
ECU	Ecuador	0.676	0.599
EGY	Egypt	0.000	0.000
SLV	El Salvador	0.003	0.017
GNQ	Equatorial Guinea	0.116	0.036
ERI	Eritrea	0.831	0.790
EST	Estonia	0.001	0.002

Table S10. *Cont.*

ISO3	Country Name	Urban Per Capita Drought Exposure	Rural Per Capita Drought Exposure
ETH	Ethiopia	0.611	0.544
FRO	Faeroe Islands	0.000	0.000
FLK	Falkland Islands	0.000	0.001
FJI	Fiji	0.262	0.234
FIN	Finland [4]	0.011	0.018
FRA	France	0.038	0.047
GUF	French Guiana	0.000	0.000
PYF	French Polynesia	0.000	0.066
GAB	Gabon	0.091	0.076
GMB	Gambia	0.412	0.436
GEO	Georgia	0.010	0.036
DEU	Germany	0.053	0.045
GHA	Ghana	0.058	0.173
GIB	Gibraltar	1.000	0.000
GRC	Greece	0.561	0.489
GRL	Greenland	0.049	0.014
GRD	Grenada	0.000	0.000
GLP	Guadeloupe [5]	0.408	0.400
GUM	Guam	0.400	0.400
GTM	Guatemala	0.880	0.804
GIN	Guinea	0.515	0.273
GNB	Guinea-Bissau	0.226	0.299
GUY	Guyana	0.200	0.190
HTI	Haiti	0.067	0.047
HND	Honduras	0.065	0.070
HKG	Hong Kong [6]	0.095	0.000
HUN	Hungary	0.026	0.027
ISL	Iceland	0.000	0.000
IND	India	0.652	0.653
IDN	Indonesia	0.424	0.426
IRN	Iran	0.659	0.671
IRQ	Iraq	0.712	0.735
IRL	Ireland	0.004	0.005
IMN	Isle of Man	0.000	0.000
ISR	Israel	0.335	0.467
ITA	Italy	0.083	0.095
JAM	Jamaica	0.016	0.040
JPN	Japan	0.009	0.011
JOR	Jordan	0.875	0.817
KAZ	Kazakhstan	0.465	0.392
KEN	Kenya	0.253	0.319
KIR	Kiribati	0.700	0.715
KWT	Kuwait	0.000	0.046

Table S10. *Cont.*

ISO3	Country Name	Urban Per Capita Drought Exposure	Rural Per Capita Drought Exposure
KGZ	Kyrgyzstan	0.482	0.436
LAO	Laos	0.487	0.551
LVA	Latvia	0.000	0.001
LBN	Lebanon	0.900	0.900
LSO	Lesotho	0.686	0.807
LBR	Liberia	0.169	0.094
LBY	Libya	0.441	0.417
LIE	Liechtenstein	0.000	0.000
LTU	Lithuania	0.000	0.000
LUX	Luxembourg	0.002	0.008
MAC	Macao [6]	0.100	0.000
MKD	Macedonia	0.009	0.034
MDG	Madagascar	0.199	0.257
MWI	Malawi	0.727	0.697
MYS	Malaysia	0.029	0.047
MDV	Maldives	0.000	0.000
MLI	Mali	0.412	0.358
MLT	Malta	0.900	0.897
MHL	Marshall Islands	0.000	0.000
MTQ	Martinique	0.000	0.000
MRT	Mauritania	0.185	0.163
MUS	Mauritius	0.000	0.042
MEX	Mexico	0.532	0.511
FSM	Micronesia, Federated States of	0.395	0.401
MDA	Moldova, Republic of	0.154	0.122
MCO	Monaco	0.100	0.000
MNG	Mongolia	0.328	0.078
MNE	Montenegro	0.000	0.000
MSR	Montserrat	0.637	0.361
MAR	Morocco [7]	0.700	0.661
MOZ	Mozambique	0.588	0.627
MMR	Myanmar	0.851	0.847
NAM	Namibia	0.646	0.558
NRU	Nauru	0.900	0.000
NPL	Nepal	0.575	0.503
NLD	Netherlands	0.056	0.069
ANT	Netherlands Antilles	0.426	0.415
NCL	New Caledonia	0.400	0.419
NZL	New Zealand	0.017	0.013
NIC	Nicaragua	0.634	0.306
NER	Niger	0.411	0.388
NGA	Nigeria	0.465	0.437
NIU	Niue	0.187	0.208

Table S10. *Cont.*

ISO3	Country Name	Urban Per Capita Drought Exposure	Rural Per Capita Drought Exposure
PRK	North Korea [8]	0.218	0.227
MNP	Northern Mariana Islands	0.224	0.284
NOR	Norway	0.062	0.046
OMN	Oman	0.011	0.025
PAK	Pakistan	0.787	0.750
PLW	Palau	0.392	0.400
PSE	Palestine	0.466	0.754
PAN	Panama	0.045	0.082
PNG	Papua New Guinea	0.160	0.204
PRY	Paraguay	0.082	0.097
PER	Peru	0.165	0.190
PHL	Philippines	0.394	0.390
PCN	Pitcairn Islands	0.000	0.000
POL	Poland	0.035	0.032
PRT	Portugal	0.399	0.353
PRI	Puerto Rico	0.000	0.000
QAT	Qatar	0.000	0.000
REU	Réunion	0.000	0.000
ROU	Romania	0.047	0.040
RUS	Russia	0.098	0.132
RWA	Rwanda	0.006	0.013
SHN	Saint Helena	0.000	0.000
KNA	Saint Kitts and Nevis	0.895	0.902
LCA	Saint Lucia	0.000	0.000
SPM	Saint Pierre and Miquelon	0.000	0.000
VCT	Saint Vincent and the Grenadines	0.000	0.000
WSM	Samoa	0.099	0.092
SMR	San Marino	0.000	0.000
STP	Sao Tome and Principe	0.000	0.000
SAU	Saudi Arabia	0.021	0.056
SEN	Senegal	0.412	0.431
SRB	Serbia	0.010	0.021
SYC	Seychelles	0.000	0.000
SLE	Sierra Leone	0.671	0.536
SGP	Singapore	0.000	0.000
SVK	Slovakia	0.000	0.000
SVN	Slovenia	0.000	0.000
SLB	Solomon Islands	0.059	0.054
SOM	Somalia	0.867	0.719
ZAF	South Africa	0.514	0.522
KOR	South Korea [9]	0.166	0.149
ESP	Spain	0.404	0.485
LKA	Sri Lanka	0.051	0.108

Table S10. *Cont.*

ISO3	Country Name	Urban Per Capita Drought Exposure	Rural Per Capita Drought Exposure
SDN	Sudan [10]	0.326	0.523
SUR	Suriname	0.000	0.001
SWZ	Swaziland	0.901	0.900
SWE	Sweden	0.115	0.103
CHE	Switzerland	0.000	0.000
SYR	Syria	0.867	0.801
TJK	Tajikistan	0.680	0.609
TZA	Tanzania, United Republic of	0.325	0.407
THA	Thailand	0.483	0.598
TLS	Timor-Leste	0.512	0.524
TGO	Togo	0.154	0.287
TKL	Tokelau	0.000	0.000
TON	Tonga	0.409	0.316
TTO	Trinidad and Tobago	0.000	0.000
TUN	Tunisia	0.308	0.253
TUR	Turkey	0.369	0.434
TKM	Turkmenistan	0.112	0.169
TCA	Turks and Caicos Islands	0.000	0.000
TUV	Tuvalu	0.096	0.661
UGA	Uganda	0.013	0.057
UKR	Ukraine	0.030	0.028
ARE	United Arab Emirates	0.689	0.453
GBR	United Kingdom	0.033	0.024
USA	United States of America	0.228	0.117
VIR	United States Virgin Islands	0.498	0.493
URY	Uruguay	0.107	0.113
UZB	Uzbekistan	0.495	0.406
VUT	Vanuatu	0.363	0.248
VAT	Vatican City State [11]	0.100	0.000
VEN	Venezuela	0.402	0.318
VNM	Vietnam	0.372	0.382
WLF	Wallis and Futuna Islands	0.000	0.200
YEM	Yemen	0.000	0.001
ZMB	Zambia	0.655	0.592
ZWE	Zimbabwe	0.878	0.773

Notes: [1] includes Christmas Island and Cocos islands; [2] includes Guernsey and Jersey; [3] includes Mayotte; [4] includes Aland Islands; [5] includes Saint Barthelemy and Saint Martin; [6] Special Administrative Region (SAR) of China; [7] includes Western Sahara; [8] Democratic People's Republic of Korea; [9] Republic of Korea; [10] includes Southern Sudan; [11] Holy See.

Table S11. Urban and rural population flood exposure.

ISO3	Country Name	Urban Per Capita Flood Exposure	Rural Per Capita Flood Exposure
AFG	Afghanistan	0.725	0.518
ALB	Albania	0.159	0.192
DZA	Algeria	0.434	0.403
ASM	American Samoa	0.000	0.000
AND	Andorra	0.100	0.101
AGO	Angola	0.147	0.051
AIA	Anguilla	0.037	0.000
ATG	Antigua and Barbuda	0.099	0.099
ARG	Argentina	0.657	0.451
ARM	Armenia	0.018	0.063
ABW	Aruba	0.102	0.098
AUS	Australia [1]	0.435	0.222
AUT	Austria	0.423	0.381
AZE	Azerbaijan	0.072	0.084
BHS	Bahamas	0.000	0.000
BHR	Bahrain	0.000	0.001
BGD	Bangladesh	0.999	0.999
BRB	Barbados	0.101	0.098
BLR	Belarus	0.157	0.222
BEL	Belgium	0.534	0.593
BLZ	Belize	0.396	0.414
BEN	Benin	0.421	0.453
BMU	Bermuda	0.100	0.000
BTN	Bhutan	0.525	0.532
BOL	Bolivia	0.649	0.562
BIH	Bosnia and Herzegovina	0.272	0.267
BWA	Botswana	0.070	0.098
BRA	Brazil	0.610	0.315
VGB	British Virgin Islands	0.000	0.000
BRN	Brunei	0.000	0.006
BGR	Bulgaria	0.058	0.082
BFA	Burkina Faso	0.324	0.282
BDI	Burundi	0.498	0.572
KHM	Cambodia	0.844	0.879
CMR	Cameroon	0.241	0.141
CAN	Canada	0.083	0.061
CPV	Cape Verde	0.000	0.000
CYM	Cayman Islands	0.000	0.000
CAF	Central African Republic	0.442	0.226
TCD	Chad	0.242	0.233
CHA	Channel Islands [2]	0.000	0.000
CHL	Chile	0.670	0.355
CHN	China	0.577	0.527

Table S11. *Cont.*

ISO3	Country Name	Urban Per Capita Flood Exposure	Rural Per Capita Flood Exposure
COL	Colombia	0.855	0.843
COM	Comoros [3]	0.025	0.043
COG	Congo, Republic of the	0.229	0.145
COK	Cook Islands	0.000	0.000
CRI	Costa Rica	0.506	0.679
CIV	Côte d'Ivoire	0.045	0.022
HRV	Croatia	0.103	0.125
CUB	Cuba	0.467	0.647
CYP	Cyprus	0.000	0.000
CZE	Czech Republic	0.539	0.496
COD	Democratic Republic of the Congo	0.275	0.180
DNK	Denmark	0.000	0.000
DJI	Djibouti	0.015	0.117
DMA	Dominica	0.096	0.097
DOM	Dominican Republic	0.796	0.786
ECU	Ecuador	0.854	0.800
EGY	Egypt	0.065	0.015
SLV	El Salvador	0.900	0.897
GNQ	Equatorial Guinea	0.000	0.000
ERI	Eritrea	0.000	0.049
EST	Estonia	0.000	0.000
ETH	Ethiopia	0.269	0.222
FRO	Faeroe Islands	0.000	0.000
FLK	Falkland Islands	0.000	0.000
FJI	Fiji	0.334	0.285
FIN	Finland [4]	0.000	0.000
FRA	France	0.550	0.423
GUF	French Guiana	0.000	0.000
PYF	French Polynesia	0.000	0.000
GAB	Gabon	0.030	0.049
GMB	Gambia	0.000	0.000
GEO	Georgia	0.378	0.331
DEU	Germany	0.602	0.582
GHA	Ghana	0.325	0.187
GIB	Gibraltar	0.000	0.000
GRC	Greece	0.289	0.138
GRL	Greenland	0.000	0.000
GRD	Grenada	0.000	0.000
GLP	Guadeloupe [5]	0.355	0.325
GUM	Guam	0.100	0.100
GTM	Guatemala	0.973	0.936
GIN	Guinea	0.009	0.018
GNB	Guinea-Bissau	0.000	0.000

Table S11. *Cont.*

ISO3	Country Name	Urban Per Capita Flood Exposure	Rural Per Capita Flood Exposure
GUY	Guyana	0.004	0.022
HTI	Haiti	0.912	0.830
HND	Honduras	0.905	0.884
HKG	Hong Kong [6]	0.997	0.000
HUN	Hungary	0.300	0.380
ISL	Iceland	0.000	0.000
IND	India	0.671	0.580
IDN	Indonesia	0.761	0.639
IRN	Iran	0.560	0.503
IRQ	Iraq	0.051	0.054
IRL	Ireland	0.235	0.099
IMN	Isle of Man	0.000	0.000
ISR	Israel	0.424	0.508
ITA	Italy	0.348	0.324
JAM	Jamaica	0.980	0.951
JPN	Japan	0.461	0.291
JOR	Jordan	0.281	0.331
KAZ	Kazakhstan	0.023	0.052
KEN	Kenya	0.880	0.805
KIR	Kiribati	0.000	0.000
KWT	Kuwait	0.000	0.024
KGZ	Kyrgyzstan	0.085	0.087
LAO	Laos	0.547	0.523
LVA	Latvia	0.000	0.000
LBN	Lebanon	0.607	0.552
LSO	Lesotho	0.168	0.299
LBR	Liberia	0.001	0.003
LBY	Libya	0.000	0.000
LIE	Liechtenstein	0.755	0.924
LTU	Lithuania	0.013	0.008
LUX	Luxembourg	0.768	0.768
MAC	Macao [6]	1.000	0.000
MKD	Macedonia	0.445	0.433
MDG	Madagascar	0.372	0.238
MWI	Malawi	0.472	0.432
MYS	Malaysia	0.685	0.638
MDV	Maldives	0.000	0.000
MLI	Mali	0.121	0.114
MLT	Malta	0.000	0.000
MHL	Marshall Islands	0.000	0.000
MTQ	Martinique	0.015	0.057
MRT	Mauritania	0.014	0.029
MUS	Mauritius	0.000	0.000

Table S11. *Cont.*

ISO3	Country Name	Urban Per Capita Flood Exposure	Rural Per Capita Flood Exposure
MEX	Mexico	0.552	0.497
FSM	Micronesia, Federated States of	0.000	0.000
MDA	Moldova, Republic of	0.149	0.174
MCO	Monaco	0.400	0.000
MNG	Mongolia	0.002	0.004
MNE	Montenegro	0.248	0.226
MSR	Montserrat	0.000	0.000
MAR	Morocco [7]	0.168	0.128
MOZ	Mozambique	0.537	0.364
MMR	Myanmar	0.081	0.092
NAM	Namibia	0.000	0.000
NRU	Nauru	0.000	0.000
NPL	Nepal	0.983	0.948
NLD	Netherlands	0.271	0.293
ANT	Netherlands Antilles	0.001	0.032
NCL	New Caledonia	0.004	0.008
NZL	New Zealand	0.474	0.270
NIC	Nicaragua	0.688	0.675
NER	Niger	0.707	0.688
NGA	Nigeria	0.343	0.305
NIU	Niue	0.000	0.000
PRK	North Korea [8]	0.354	0.426
MNP	Northern Mariana Islands	0.000	0.000
NOR	Norway	0.004	0.005
OMN	Oman	0.011	0.014
PAK	Pakistan	0.566	0.472
PLW	Palau	0.000	0.000
PSE	Palestine	0.358	0.442
PAN	Panama	0.432	0.371
PNG	Papua New Guinea	0.024	0.017
PRY	Paraguay	0.874	0.798
PER	Peru	0.655	0.643
PHL	Philippines	0.832	0.711
PCN	Pitcairn Islands	0.000	0.000
POL	Poland	0.306	0.326
PRT	Portugal	0.336	0.388
PRI	Puerto Rico	0.557	0.578
QAT	Qatar	0.000	0.002
REU	Réunion	0.000	0.000
ROU	Romania	0.648	0.674
RUS	Russia	0.109	0.093
RWA	Rwanda	0.809	0.820
SHN	Saint Helena	0.000	0.000

Table S11. *Cont.*

ISO3	Country Name	Urban Per Capita Flood Exposure	Rural Per Capita Flood Exposure
KNA	Saint Kitts and Nevis	0.000	0.000
LCA	Saint Lucia	0.091	0.042
SPM	Saint Pierre and Miquelon	0.000	0.000
VCT	Saint Vincent and the Grenadines	0.000	0.000
WSM	Samoa	0.099	0.059
SMR	San Marino	0.100	0.102
STP	Sao Tome and Principe	0.000	0.000
SAU	Saudi Arabia	0.134	0.055
SEN	Senegal	0.090	0.068
SRB	Serbia	0.546	0.499
SYC	Seychelles	0.000	0.000
SLE	Sierra Leone	0.034	0.059
SGP	Singapore	0.900	0.000
SVK	Slovakia	0.484	0.505
SVN	Slovenia	0.055	0.078
SLB	Solomon Islands	0.052	0.060
SOM	Somalia	0.632	0.415
ZAF	South Africa	0.352	0.478
KOR	South Korea [9]	0.874	0.819
ESP	Spain	0.214	0.154
LKA	Sri Lanka	0.803	0.894
SDN	Sudan [10]	0.495	0.229
SUR	Suriname	0.000	0.001
SWZ	Swaziland	0.196	0.358
SWE	Sweden	0.001	0.001
CHE	Switzerland	0.749	0.757
SYR	Syria	0.261	0.219
TJK	Tajikistan	0.671	0.683
TZA	Tanzania, United Republic of	0.509	0.407
THA	Thailand	0.765	0.841
TLS	Timor-Leste	0.135	0.209
TGO	Togo	0.150	0.296
TKL	Tokelau	0.000	0.000
TON	Tonga	0.000	0.000
TTO	Trinidad and Tobago	0.627	0.657
TUN	Tunisia	0.267	0.383
TUR	Turkey	0.289	0.169
TKM	Turkmenistan	0.156	0.102
TCA	Turks and Caicos Islands	0.000	0.000
TUV	Tuvalu	0.000	0.000
UGA	Uganda	0.754	0.666
UKR	Ukraine	0.134	0.185
ARE	United Arab Emirates	0.000	0.002

Table S11. *Cont.*

ISO3	Country Name	Urban Per Capita Flood Exposure	Rural Per Capita Flood Exposure
GBR	United Kingdom	0.817	0.717
USA	United States of America	0.520	0.514
VIR	United States Virgin Islands	0.001	0.010
URY	Uruguay	0.497	0.625
UZB	Uzbekistan	0.310	0.299
VUT	Vanuatu	0.127	0.104
VAT	Vatican City State [11]	0.100	0.000
VEN	Venezuela	0.635	0.516
VNM	Vietnam	0.877	0.878
WLF	Wallis and Futuna Islands	0.000	0.000
YEM	Yemen	0.625	0.606
ZMB	Zambia	0.007	0.027
ZWE	Zimbabwe	0.132	0.194

Notes: [1] includes Christmas Island and Cocos islands; [2] includes Guernsey and Jersey; [3] includes Mayotte; [4] includes Aland Islands; [5] includes Saint Barthelemy and Saint Martin; [6] Special Administrative Region (SAR) of China; [7] includes Western Sahara; [8] Democratic People's Republic of Korea; [9] Republic of Korea; [10] includes Southern Sudan; [11] Holy See.

References

1. Exposure Assessment Global Model. ArcGIS Toolbox. The Water Institute, Gillings School of Global Public Health, University of North Carolina: Chapel Hill, NC, USA. Available online: http://waterinstitute.unc.edu (accessed on 17 February 2014).
2. Dilley, M.; Chen, R.S.; Deichmann, U.; Lerner-Lam, A.L.; Arnold, M.; Agwe, J.; Buys, P.; Kjekstad, O.; Lyon, B.; Yetman, G. *Natural Disaster Hotspots A Global Risk Analysis*; the International Bank for Reconstruction and Development, the World Bank and Columbia University: Washington, DC, USA, 2005.
3. *Global Assessment Report on Disaster Risk Reduction*; United Nations International Strategy for Disaster Reduction (UNISDR): Geneva, Switzerland, 2009.
4. *Global Assessment Report on Disaster Risk Reduction*; United Nations International Strategy for Disaster Reduction (UNISDR): Geneva, Switzerland, 2013.

© 2014 by the authors; licensee MDPI, Basel, Switzerland. This article is an open access article distributed under the terms and conditions of the Creative Commons Attribution license (http://creativecommons.org/licenses/by/3.0/).
